# Interpretable abstractions of artificial neural networks predict behavior and neural activity during human information gathering

**DOI:** 10.1038/s41593-026-02342-9

**Published:** 2026-06-26

**Authors:** Simone D’Ambrogio, Jan Grohn, Nima Khalighinejad, Marcelo G. Mattar, Laurence Hunt, Matthew F. S. Rushworth

**Affiliations:** 1 Department of Experimental Psychology, University of Oxford, Oxford, UK; 2 Department of Psychology, New York University, New York, NY USA; 3 Nuffield Department of Clinical Neurosciences, University of Oxford, Oxford, UK

**Keywords:** Decision, Network models

## Abstract

Humans and other animals are driven to acquire information about opportunities in their environments, yet how they evaluate what is worth learning remains unclear. Here we combine artificial neural networks with symbolic regression to extract an expressive yet interpretable model that specifies how human participants evaluate decision-relevant information during choice. The recovered function depends primarily on the relative evidence accumulated across options rather than absolute uncertainty about each, revealing that participants seek information symmetry across alternatives rather than minimizing uncertainty option by option. This account outperforms standard models of uncertainty-based exploration and generalizes to an independent dataset. Using ultrahigh-field (7T) functional magnetic resonance imaging optimized for midbrain and brainstem, we simultaneously measured activity across five neuromodulatory nuclei and two cortical regions. Ventral tegmental area activity showed opposed coding of information and selection values, a pattern suited to arbitrating between sampling and choosing, and anterior cingulate cortex and anterior insula tracked value-of-information computations.

## Abstract (web summary)

D’Ambrogio et al. combine deep learning and symbolic regression to report an interpretable equation of how humans value information. The equation predicts choices and neural activity in anterior insula, cingulate cortex and midbrain nuclei.

## Main

**Fig. 1:**
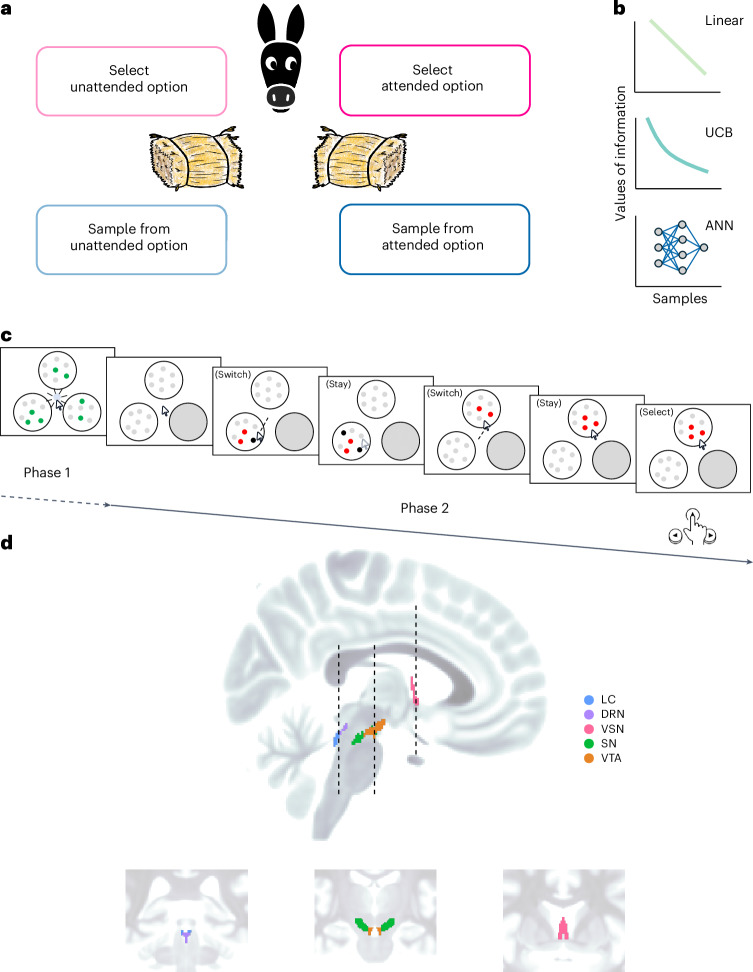
Information-sampling task. **a**, The decision-making process involves two key decisions: whether to gather more information or make a selection (top versus bottom) and, if gathering information, whether to sample from the currently attended option or switch to the alternative (top right versus top left). **b**, Three approaches to computing the VoI as a function of the number of samples. Top: a linear function where value decreases at a constant rate with each additional sample. Middle: UCB algorithm that captures diminishing returns, with steeper initial decline that flattens as samples accumulate. Bottom: an ANN that learns the mapping between samples and information value; the learned function’s form is not specified a priori. **c**, Task structure showing the two phases of the information-sampling task. In phase 1, participants are presented with three patches of dots covered by green or gray covers. After revealing the green-covered dots in each patch, one patch is blocked (gray circle). In phase 2, participants can freely sample information by hovering over patches, with gray-covered dots revealing their true colors sequentially, before making a final selection. When participants switched patches, previously revealed dots in the unattended patch returned to their gray-covered state, requiring reliance on memory (as illustrated by the gray patch in phase 2, right panel). **d**, Brain ROIs: LC, DRN, VSN, SN and VTA, which have been implicated in uncertainty processing and information sampling.

Decisions should be based on evidence. Once sufficient evidence has been sampled, the agent can decide which option to select (Fig. 1a, top). But in addition to guiding the choice, evidence should also simultaneously be used to evaluate whether there is sufficient information to warrant a decision or whether further information must be sampled (Fig. 1a, bottom)^1–4^. When sampling information, attentional constraints mean that decision-makers typically focus on only one option at a time^5^. There is therefore a further decision as to whether more evidence should be gathered from the currently attended option or whether attention should be shifted to an alternative (Fig. 1a, bottom). Gathering more information can improve decision quality; however, it also comes at the expense of time, energy and lost opportunities to engage in other activities. An extreme illustration is the fourteenth-century thought experiment of Buridan’s ass (Fig. 1a)^6^: unable to choose between two equal piles of hay, the ass starves. Although several studies have shed light on the computational and neural mechanisms underlying value learning and option selection^7,8^, the factors that determine when to stop gathering information and commit to a final decision, as well as which options to sample from, remain an active area of investigation^1,4,9–11^.

A standard approach in cognitive neuroscience is to formalize hypotheses about cognitive computations as mathematical models that generate quantitative predictions for behavior and neural activity. Two such hypotheses can be considered for how individuals guide their information sampling. The first posits that people compute the value of gathering additional information as a linear function of an option’s uncertainty (for example, the amount of missing knowledge; Fig. 1b, top)^12–14^. This approach would prioritize further sampling from more uncertain options. However, theories of sequential sampling processes and Bayesian updating indicate that repeatedly sampling from the same option yields diminishing returns (Fig. 1b, middle), motivating a second hypothesis: that the value of information (VoI) is computed using a nonlinear function of uncertainty^1,9^. The upper confidence bound (UCB) algorithm, a widely used exploration heuristic that captures this nonlinear relation, can formalize this hypothesis. Both linear and UCB models aim to characterize the functional form of a cognitive computation, such as the value of sampling, in a psychologically interpretable way.

Although the concept of diminishing returns helps narrow down the space of hypotheses, there is still a wide range of nonlinear functions that could describe how people value information. This presents a challenge in psychology and neuroscience, where it is often difficult to select a specific instantiation of a general principle, slowing the pace of new discoveries. An alternative approach is to use machine learning to provide a more flexible, data-driven model of participants’ choices: for example, using artificial neural networks (ANNs) to fit behavior. ANNs can learn complex mappings from data, vastly expanding the space of candidate functions that can be considered (Fig. 1b, bottom)^15,16^. The universal function approximation theorem underpins this method, asserting that sufficiently deep neural networks can model any continuous function to arbitrary precision, given sufficient training data. This capability allows us to learn directly from data how individuals might compute the VoI to guide their behavior, without needing to specify the exact form of the function in advance. By comparing an ANN’s performance against established, fixed-form functions (like linear or UCB), we can directly assess whether these more constrained models adequately capture the complexities of information valuation. ANNs can thus be used as a tool to discover a potentially more accurate functional description of how people assign value to information. However, this expressive power comes at a cost. Although ANNs may yield more accurate predictions, they typically lack interpretability. The learned representations are distributed, high-dimensional and opaque, making it difficult to extract mechanistic or symbolic insights about underlying cognitive processes.

Here we propose a modeling approach that yields expressive yet interpretable models of choice. First, we integrate data-driven and knowledge-driven components into a hybrid model of subjects’ information-sampling and decision-making behavior^15^. The knowledge-driven component incorporates established cognitive principles, such as the mechanisms underlying option selection^8,17^. The data-driven component utilizes ANNs to model aspects of cognition that are difficult to define, such as complex information-sampling strategies. This hybrid is more expressive than a fully knowledge-driven model and more interpretable than a fully data-driven one. Second, we apply symbolic regression^18,19^ to the trained ANN to recover a compact, four-parameter approximation of its learned function, providing a new route to theory generation from data. We show that the recovered function reveals an information-symmetry principle (participants value sampling based on the relative evidence accumulated across options rather than option-wise uncertainty) and generalizes to an entirely separate experimental dataset on human information sampling^20^.

We used our approach to model human behavioral data from a task designed to assess evaluation of information and choice selection (Fig. 1c). Several brain regions are thought to play key roles in information sampling under uncertainty. Notably, all the neuromodulatory systems with their origins in the ventral tegmental area (VTA), substantia nigra (SN), dorsal raphe nucleus (DRN), locus coeruleus (LC) and ventral septal nucleus (VSN) have at one time or another been proposed to reflect uncertainty or the potential for information gain^3,21–27^ (Fig. 1d). It is less clear whether each neuromodulatory system has a specific or unique relationship with uncertainty. Recording simultaneously from multiple nuclei has been difficult in animal models, and conventional human neuroimaging lacks the spatial resolution to reliably isolate their signals. Here we exploit high-resolution (1-mm isotropic), rapid-repetition-time (1.378 s), accelerated ultrahigh-field (7T) functional magnetic resonance imaging (fMRI)^27,28^ to measure activity simultaneously across all five neuromodulatory nuclei and two interconnected cortical regions that project to them: the anterior insula (AI) and anterior cingulate cortex (ACC)^3,22,29–33^. Guided by the hybrid-ANN model, we identified distinct patterns across these seven regions: VTA activity arbitrated between information gathering and choice, and ACC and AI tracked VoI signals that guided sampling behavior.

## Results

### Sampling behavior adaptively scales with task difficulty and uncertainty

Twenty participants completed an information-sampling task (Fig. 1c) inside a 7T MRI scanner. In each trial, they were presented with two patches of 100 moving dots. Participants were informed that the true color of each dot was either red or black, and the goal was to select the patch with the highest number of red dots. At the start of each trial, however, the true color of the dots in each patch was unknown because they were hidden under green or gray covers. The number of green dot covers (revealed simultaneously upon first hover) varied from 5 to 30, and the number of gray dot covers (revealed sequentially) correspondingly ranged from 70 to 95. Participants used a trackball to hover over a patch, and this led to the true color (red or black) being gradually revealed.

Upon hovering, participants had to wait 2 s before any new information appeared. After the waiting period, the green dot covers were all removed to reveal which dots were either red or black. The gray covers of individual dots were then also removed, but they were removed one by one every 150 ms, again revealing either a red or black dot. Each patch, therefore, provided a different amount of initial information on sampling, indicated by green dots, which varied between trials. For instance, if a patch had 20 green dots, the color of these dots would be revealed as red or black during the first sample of the first visit to that patch. The gray dots in the patch were then revealed as either red or black one by one. By manipulating the amount of initial information gain, this design allowed us to separate the time spent in a patch from the uncertainty about the number of red dots in that patch.

This design also allowed us to examine how background uncertainty, as well as the uncertainty of the options themselves, affects sampling behavior. By signaling the initial amount of information with green dots, participants could estimate the starting uncertainty of each option. Once all green dots were shown, one of the three options was blocked, leaving only two options available. The blocked option’s uncertainty (background uncertainty) was unaffected by participants’ actions and did not impact the task of selecting the patch with more red dots. Each participant completed four sessions, each of which lasted 25 min. Participants were instructed and incentivized to make as many correct choices as possible in each session. Notably, because each session had a fixed duration, spending excessive time revealing all dots in a single trial reduced the number of subsequent trials participants could attempt, thereby limiting their overall potential rewards.

**Fig. 2:**
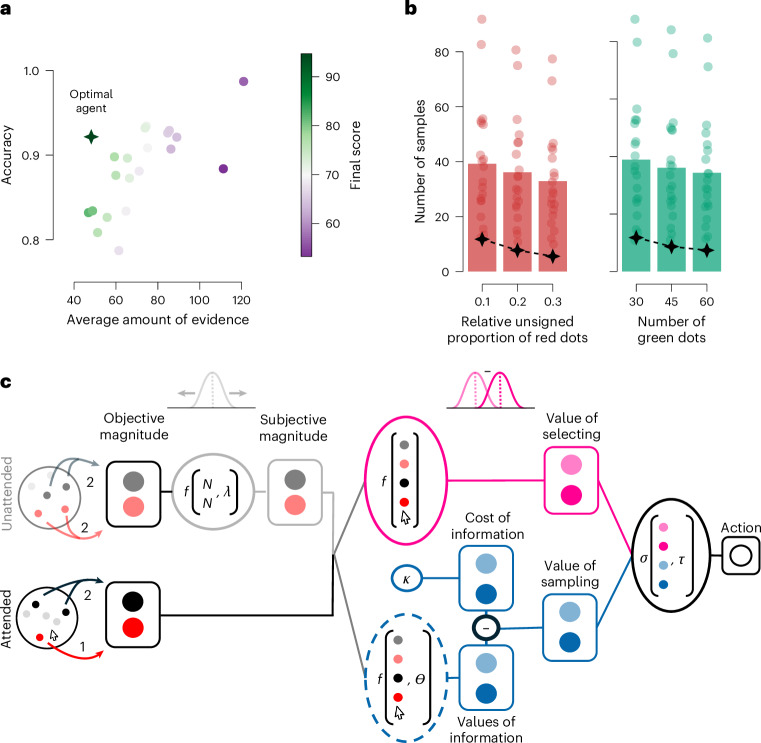
Sampling behavior and computational model. **a**, Relationship between accuracy and average amount of evidence collected. Each colored dot represents an individual participant, with color indicating their final score according to the color scale on the right. The dark green star marks the position of the optimal agent (optimal under the cost assumptions specified in Methods). *n* = 20 participants. **b**, Number of samples collected under different task conditions. Left: bar graph showing the number of samples collected across three levels of relative unsigned proportion of red dots between patches (0.1, 0.2, 0.3). Right: bar graph showing the number of samples collected across three levels of initial information (30, 45, 60 green dots). Individual participant data points are shown as colored dots, and black stars indicate the model-derived optimal agent’s behavior. **c**, Schematic of the computational model. The model transforms objective magnitudes (number and color of dots in each patch) into subjective magnitudes through attentional discounting and memory decay functions. These subjective magnitudes are used to compute the value of selecting each patch (pink) and the value of sampling more information (blue), which together determine the final action. The dotted blue circle highlights the component that computes the VoI, which is implemented using a linear function, UCB algorithm or ANN. Source data

Participants exhibited varying preferences for speed versus accuracy in their sampling behavior (Fig. 2a). Some participants opted to spend more time gathering information, aiming to increase accuracy, whereas others prioritized faster decisions, accepting a higher risk of error (correlation between amount of information and accuracy: *r* = 0.74, *t* = 4.33, *P* = 4.00 × 10^−4^). As noted, each trial differed in two key aspects: the initial uncertainty of each option, as indicated by the green dots, and the final difference in red dots between the patches, ranging from a difference of 30 (easy trials) to 10 (difficult trials). Participants tended to gather more samples when the initial uncertainty was higher (there were fewer green dots, which were uncovered at the beginning of the sampling period, and more gray dots, which were uncovered only one by one during sampling: *β* = −0.069, standard error (*s*.*e*.) = 0.013, *z* = −5.18, *P* = 2.27 × 10^−7^) and when the final discrepancy in red dots was smaller (*β* = −0.131, *s*.*e*. = 0.019, *z* = −6.87, *P* = 6.48 × 10^−12^; Fig. 2b). To assess whether this pattern is adaptive, we estimated an optimal policy for this task, given specific assumptions about sampling costs (Methods), solving the Bellman equation using dynamic programming^34,35^. We simulated action sequences from this optimal agent and compared them to the actual action sequences exhibited by participants. We found that participants’ sampling behavior qualitatively resembled the optimal policy derived from our computational analysis: they adaptively increased their sampling time when initial uncertainty (100 − number of green dots) was higher or when decisions were more challenging (lower disparity between the number of red dots associated with each option). Some participants deviated from this policy by sampling more information than was optimal; although their accuracy generally increased, they tended ultimately to earn fewer points (Fig. 2a) because the extra time spent per trial reduced the number of trials they could complete.

Finally, we examined which kinds of uncertainty influenced participants’ sampling behavior. In our task design, participants knew the initial uncertainty (number of green dots) of the blocked option, which should be irrelevant for optimal sampling between the two available options, as well as the uncertainty of the decision-relevant options that were available to be chosen. We tested whether this irrelevant uncertainty affected participants’ sampling behavior using a mixed-effects Poisson regression model. It did not. The analysis revealed that participants sampled less from the attended option when this attended option had more initial green dots (*β* = −0.138, *s*.*e*. = 0.017, *z* = −8.11, *P* = 5.24 × 10^−16^). They also sampled more from the attended option when the other option, the unattended option, had more initial green dots (*β* = 0.173, *s*.*e*. = 0.024, *z* = 7.19, *P* = 6.42 × 10^−13^). Crucially, however, the number of green dots in the blocked option did not significantly influence sampling behavior (*β* = −0.006, *s*.*e*. = 0.006, *z* = −1.01, *P* = 0.310), suggesting that participants were able to appropriately ignore task-irrelevant background uncertainty even when their behavior was influenced by task-relevant uncertainty. This pattern held true even immediately after exposure to the blocked option (no significant interaction between blocked dots and first visit, *β* = 0.011, *s*.*e*. = 0.008, *z* = 1.41, *P* = 0.158) and remained consistent across sessions, indicating that participants maintained this optimal strategy throughout the experiment. Consequently, this background uncertainty will not be the focus of our subsequent modeling and neural analyses, which are restricted to trials where two options were available. These results suggest that participants were able to distinguish between relevant and irrelevant sources of uncertainty in their sampling behavior.

### The ANN-derived VoI predicts participants’ sampling decisions

To investigate participants’ strategies, we developed and fitted a computational model that takes as input the current state (that is, the number of red and black dots in both patches) to compute action-specific values. We considered four actions: staying (sampling from the currently attended patch; Figs. 2c and 1a, bottom right), switching (sampling from the unattended patch; Figs. 2c and 1a, bottom left) and selecting either the attended or unattended patch (Figs. 1a, top, and 2c). To make any one of these four actions, the model maintains and updates its beliefs about the likely number of red dots in both the currently attended patch and the unattended patch. For the attended patch, this belief is updated directly based on the colors of the dots revealed during ongoing sampling.

For the unattended patch, where there is no direct sensory input, the model relies on information held in working memory: for a not-yet-visited patch, the number of green dots determines initial uncertainty and the proportion of red dots is estimated from the currently attended patch; for a previously visited patch, memory of past observations is used and is assumed to decay over time^36,37^ (Methods).

**Fig. 3:**
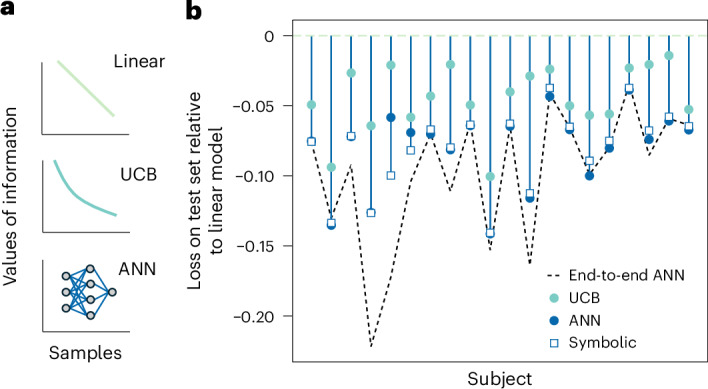
Model comparison. **a**, Schematic of three approaches to computing the VoI as a function of the number of samples (see Fig. 1b for details): linear function (top), UCB algorithm with diminishing returns (middle) and ANN with learned function form (bottom). **b**, Model comparison across participants showing loss relative to the linear model. Each vertical line represents a participant, with light blue circles (UCB model), dark blue circles (hybrid-ANN model) and open squares (symbolic model derived through symbolic regression) showing the relative loss for each model type. The dotted line shows the performance of an end-to-end ANN (trained to directly predict actions without cognitive structure). Lower values on the *y* axis indicate better model fit. *n* = 20 participants; leave-one-out cross-validation. Source data

To evaluate how participants computed the VoI, we compared three models with cross-validation. The first assumes a linear relationship between VoI and the number of collected samples (Fig. 3a, top): each new sample reduces the value of learning about the patch by the same amount. The second uses a UCB function, which assigns decreasing value as samples accumulate (Fig. 3a, middle). The third, our hybrid model, uses an ANN to learn the mapping from state variables (such as the number of revealed dots) to the value of gathering additional information (Fig. 3a, bottom), allowing for complex, nonlinear relationships without imposing a functional form a priori.

Once the ANN estimates the VoI for sampling each patch, the model computes the overall value for four potential actions (Fig. 2c, right). For the two sampling actions, the value of staying to sample the currently attended patch is its ANN-estimated VoI. The value of switching to sample the unattended patch is its ANN-estimated VoI, reduced by a fitted cost of switching. For the two selection actions, the model compares the current subjective estimates of red dots (*ρ*) in the attended and unattended patches. The value of selecting the attended patch is based on the difference in these subjective estimates (*ρ*_attended_ − *ρ*_unattended_) and correspondingly for selecting the unattended patch (*ρ*_unattended_ − *ρ*_attended_). These four action values are then scaled by a temperature parameter and transformed via a softmax function to yield the probability of choosing each action.

**Extended Data Fig. 1:**
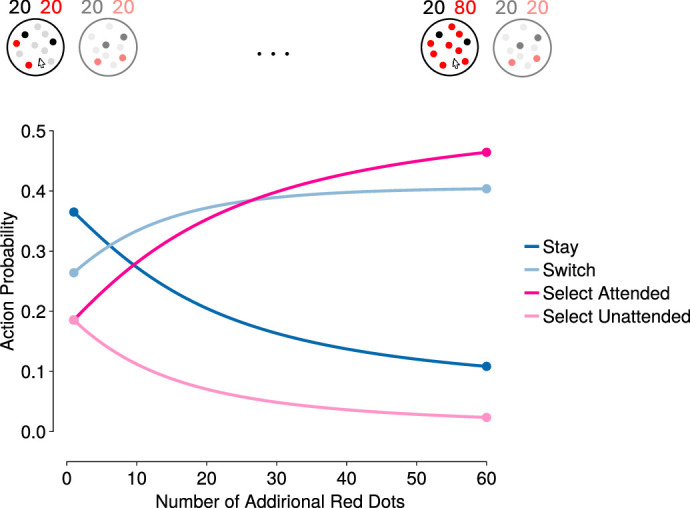
Softmax competition balances sampling and selection. Model predictions when one option becomes clearly better. Starting from equal evidence (20 red, 20 black dots in both options), we incrementally added red dots to the attended option. As the attended option became clearly better, the probability of selecting it increased (pink line), while the probability of continuing to sample (staying, dark blue line) decreased. This demonstrates that although the value of information in the hybrid model depends only on sample counts (N) rather than evidence quality (red/black composition), the model correctly predicts reduced sampling when evidence clearly favors one option: softmax competition between sampling and selection action values shifts probability mass from sampling to selection as the value of selection grows with evidence divergence. Source data

With this architecture, the model can correctly predict reduced sampling when one option becomes clearly better, even though the VoI depends only on sample counts (*N*) rather than evidence quality (red/black composition). This behavior arises from softmax competition between sampling and selection actions. When evidence strongly favors one option (large difference in red proportions), the value of selection (VoS) for that option increases substantially. In the softmax, this higher selection value reduces the probability allocated to sampling actions, even though the VoI itself remains unchanged. Thus, the balance between information seeking and choice selection emerges naturally from the interplay between these two value signals (Extended Data Fig. 1).

**Extended Data Fig. 2:**
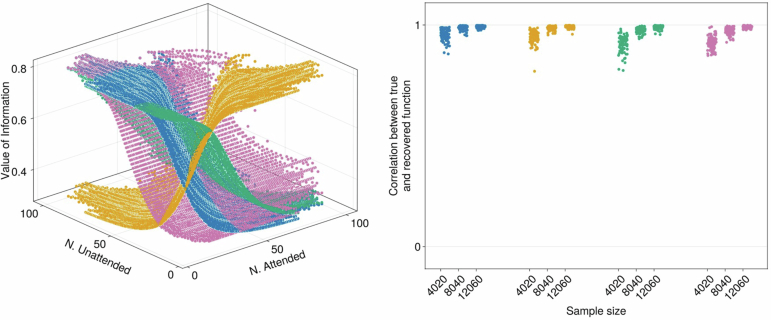
Simulation-recovery validation of the hybrid modelling approach. Data were simulated from four agents, each using a distinct non-linear value-of-information function (colour-coded blue, green, pink, and orange). Applying the hybrid modelling approach to each simulated dataset recovered the underlying generating function. Left, three-dimensional scatter showing the value of information as a function of the number of dots revealed in the attended (*N*_*attended*_) and unattended (*N*_*unattended*_) patches. Colour indicates the simulated generating function. Right, correlation between true and recovered functions across three sample sizes (4,020, 8,040, and 12,060 observations). Each dot represents a single recovery attempt; colour indicates the simulated function. Pearson r > 0.95 between recovered and true functions across sample sizes, demonstrating reliable recovery across the range of actual participant data. See Methods for simulation details. Source data

We validated the hybrid modeling approach using a simulation-recovery procedure: for data simulated from four different agents, each using a distinct nonlinear VoI function, the ANN reliably recovered the underlying function (*r* > 0.95 with the true generating function; Extended Data Fig. 2; Methods).

**Extended Data Fig. 3:**
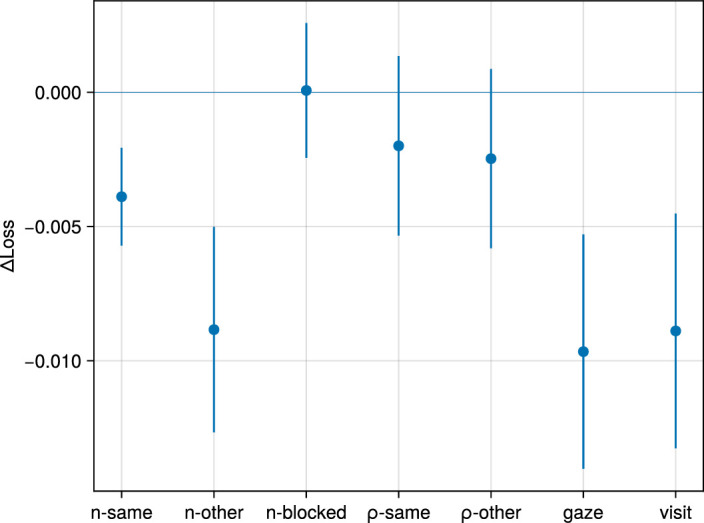
Feature selection identifies the ANN inputs essential for computing the value of information. Dot plot showing the change in model loss (ΔLoss) when each candidate feature is removed from the ANN input vector. Each blue dot indicates the mean loss difference across cross-validation folds; vertical blue lines indicate 95% confidence intervals. Features tested: N-same (non-gray dots in the attended patch), N-other (non-gray dots in the unattended patch), N-blocked (non-gray dots in the blocked patch), *ρ*-same (subjective proportion of red dots in the attended patch), *ρ*-other (subjective proportion of red dots in the unattended patch), cursor (current cursor position, that is which patch is attended), and visit (first-visit indicator: true while the participant continues sampling the first-attended patch; false after the first switch). Four features are critical—their removal substantially increases loss: cursor, visit, N-same, and N-other. In contrast, removing *ρ*-same, *ρ*-other, or N-blocked does not impair model performance. The cursor and visit variables structure how the ANN computes the value of information: cursor determines which function applies (value of staying when attending a patch, value of switching when attending the other patch), while visit modulates the parameters of these functions. n = 20 participants; leave-one-out cross-validation. Source data

We performed feature selection to identify the ANN inputs essential for computing the VoI (Extended Data Fig. 3). Four features were critical: cursor position, first-visit indicator and the number of nongray dots (*N*) in the attended and unattended patches. Notably, the subjective proportion of red dots (*ρ*) was not critical, indicating that the VoI depends on sample counts rather than decision uncertainty.

Having validated our method and identified the input features, we next compared how well each approach to computing the VoI (linear, UCB or hybrid-ANN) could account for participants’ behavior. Using a cross-validation approach, we found that the hybrid model achieved a better fit than the other two models for all participants (Fig. 3b). We also considered a symbolic model in this model comparison (Fig. 3b), which we detail in the next section. This suggests that the ANN was able to find an alternative strategy that the first two competing models did not capture. Given the ANN’s flexibility, a better fit to the data is expected; the critical question is whether this flexibility captures meaningful structure or merely overfits noise. The cross-validation approach used here, combined with the subsequent generalization tests, addresses this concern.

To benchmark our approach against a maximally expressive model, we also trained an end-to-end ANN that directly maps task states to action probabilities without imposing any cognitive structure. Such unconstrained models provide a reference bound on achievable predictive performance due to their expressive power^38^. As shown in Fig. 3b, the hybrid-ANN and symbolic models achieved levels of predictive performance similar to the end-to-end ANN (gray band shows variability across weight initializations), demonstrating that the interpretable cognitive structure we imposed captures the predictable variance in behavior.

**Extended Data Fig. 4:**
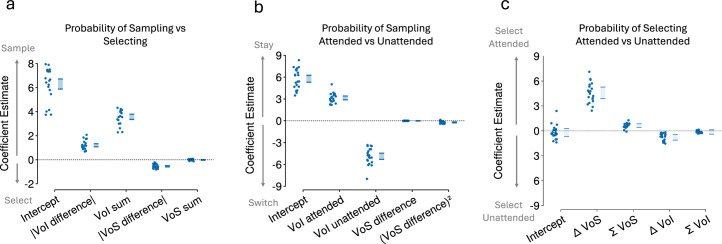
Mixed-effects logistic regressions linking the ANN-derived value of information to three nested decisions. Each panel shows subject-specific random-effect estimates (dots) together with ± 95% confidence intervals of the fixed effects (rectangles). All P-values are from two-sided Wald tests on regression coefficients. n = 20 participants. **a**, Probability of sampling versus selecting a patch. Predictors: sum of the value of information across options (VoI sum; *β* = 2.860, s.e. = 0.167, z = 17.16, P = 5.25 × 10^−66^) and unsigned difference in VoI between options (∣VoI difference∣; *β* = 1.292, s.e. = 0.085, z = 15.16, *P* = 6.14 × 10^−52^). Value-of-selection predictors (VoS sum, ∣VoS difference∣) are included as controls. **b**, Probability of staying with the currently attended patch versus switching. Predictors: VoI for the attended patch (*β* = 3.971, s.e. = 0.434, z = 9.14, P = 6.03 × 10^−20^) and VoI for the unattended patch (*β* = − 5.880, s.e. = 0.456, z = − 12.89, P = 5.13 × 10^−38^). Value-of-selection differences are included as controls. **c**, Probability of selecting the attended versus unattended patch. Predictors: difference in red-dot proportions between attended and unattended patches (*ρ* difference; *β* = 4.572, s.e. = 0.336, z = 13.60, P = 3.77 × 10^−42^); sum of red-dot proportions across patches (*ρ* sum; *β* = 0.631, s.e. = 0.104, z = 6.08, P = 1.19 × 10^−9^); and VoI(attended) - VoI(unattended) (*β* = − 0.782, s.e. = 0.159, z = − 4.91, P = 9.16 × 10^−7^). Participants were less likely to select a patch whose remaining value of information was higher than the alternative’s, a pattern consistent with an account in which participants seek to minimize uncertainty about the option they will ultimately choose. Note: the predictors used in these regressions (sums, unsigned differences, per-option VoIs) were not directly optimized during ANN training, so these regression analyses provide insight into how the learned value-of-information representation decomposes into interpretable decision variables.

We next used mixed-effects logistic regressions to examine how the ANN-derived VoI shaped each of three nested decisions in the task (Extended Data Fig. 4 and Fig. 1a): (1) whether to sample or select, (2) which patch to sample and (3) which patch to select. Participants were more likely to sample (rather than select) when the VoI was high for both options and when it differed strongly between them. Within sampling, they were more likely to stay with the currently attended patch when its VoI was high relative to the unattended option. Within selection, choices were primarily driven by the proportion of red dots, with VoI playing a secondary role. Because the specific predictors used in these regressions were not directly optimized during model training, they provide insight into how the learned VoI representation decomposes into interpretable decision variables.

Together, these analyses show that the hybrid-ANN model outperformed linear and UCB accounts (Fig. 3b) and that its VoI representation maps onto the interpretable decision variables that guide the three nested decisions in the task (Extended Data Fig. 4).

### The ANN integrates evidence from both patches to compute VoI

**Fig. 4:**
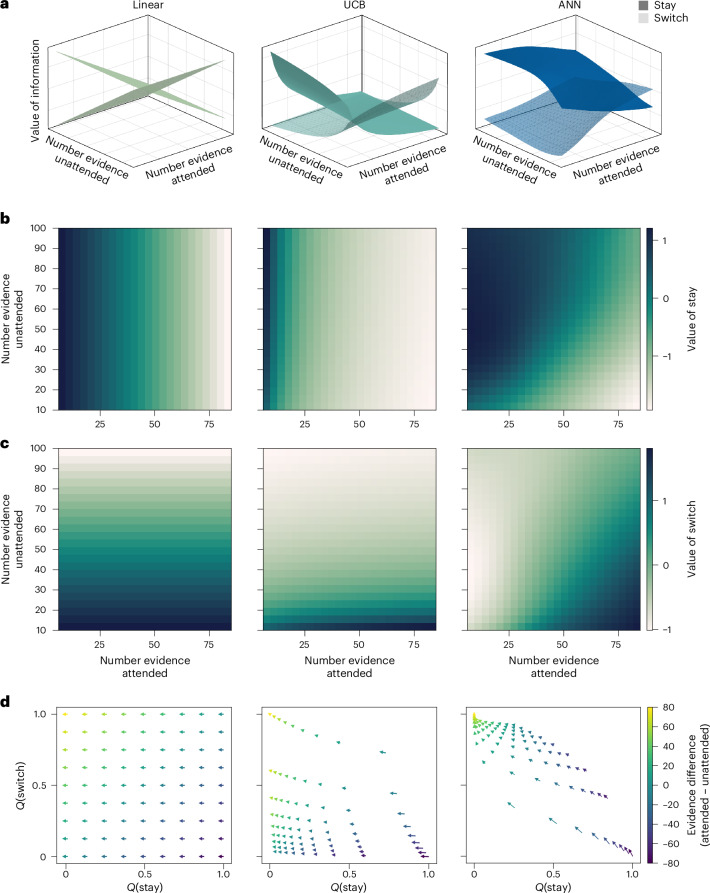
Visualization of VoI functions across computational models. **a**, 3D surface plots showing how the VoI varies with the number of revealed dots in both the attended and unattended patches. Each model (linear, UCB and ANN) is represented by two surfaces: darker color for the value of staying with the attended patch and lighter color for the value of switching to the unattended patch. **b**, Heatmaps showing the value of staying as a function of evidence collected from both patches. Color intensity represents the magnitude of the value, with the scale shown on the right. **c**, Heatmaps showing the value of switching as a function of evidence collected from both patches. **d**, Vector-field plots showing how collecting an additional sample from the attended option affects both the value of staying (*x* axis) and the value of switching (*y* axis). Each arrow represents the transition from current values (arrow origin) to updated values (arrow tip) after collecting one new sample. The color of the arrows indicates the difference in evidence between attended and unattended patches according to the color scale on the right, and the length of the arrow indicates the size of the update. Source data

Our behavioral analyses demonstrate that the hybrid-ANN approach has a better predictive performance than the linear and UCB models. To gain insight into the nature of the computation learned by the ANN, we first performed a qualitative analysis of the ANN-learned function (Fig. 4).

We visualized the learned VoI function as a surface in three-dimensional (3D) space relating the number of revealed dots in the attended and unattended patches to the values of staying and switching (Fig. 4a and heatmaps in Fig. 4b,c). The three models differ qualitatively in how they integrate cross-patch evidence. The linear model’s value of staying varies exclusively with attended evidence (and its value of switching exclusively with unattended evidence), reflecting its per-patch construction. The UCB model shows the same predominant one-dimensional dependence, with mild modulation by the other patch. In contrast, the ANN integrates evidence from both patches when computing either value: the value of staying increases with attended evidence while simultaneously decreasing with unattended evidence, producing a diagonal gradient (Fig. 4b, right). A similar two-dimensional integration emerges for the value of switching (Fig. 4c).

**Extended Data Fig. 5:**
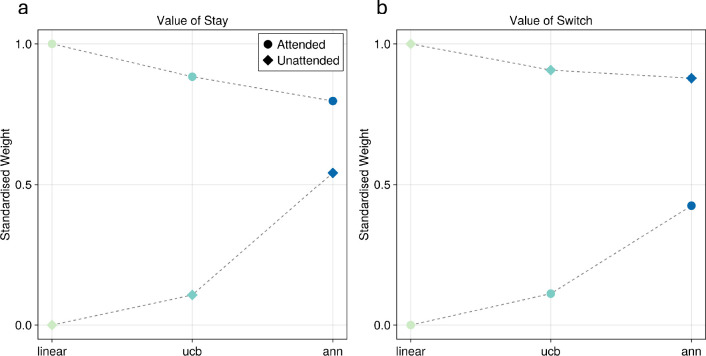
Model comparison for evidence weighting. Standardised regression coefficients from regressing each model’s output on the number of dots revealed in the attended and unattended patches. Circles represent weights for the attended evidence, diamonds represent weights for the unattended evidence; colours transition from light green to dark blue across models, and dotted lines connect the weights across models **a**, Value of staying. The linear model depends exclusively on attended evidence (*β*_attended_ = 1.0, *β*_unattended_ = 0.0). The UCB model is predominantly attended-driven with modest unattended modulation (*β*_attended_ = 0.8, *β*_unattended_ = 0.2). The ANN shows balanced weights on both dimensions (*β*_attended_ = 0.7, *β*_unattended_ = 0.6), quantitatively confirming the diagonal gradient visible in Fig. 4b**b**, Value of switching. A mirror pattern emerges, using the same visual conventions as in panel a: the linear model depends exclusively on unattended evidence, UCB predominantly on unattended with modest attended modulation, and the ANN shows balanced weighting across both dimensions. Source data

A linear regression quantifying each model’s dependence on attended versus unattended evidence confirmed this pattern: the ANN showed balanced weights (*β*_attended_ = 0.7, *β*_unattended_ = 0.6) compared with the strongly asymmetric weights of the linear and UCB models (Extended Data Fig. 5).

To visualize the dynamic consequence of this integration, we plotted how collecting one additional sample from the attended patch updates both values (Fig. 4d). For the linear and UCB models, these update arrows are predominantly horizontal: new attended evidence strongly changes the value of staying but leaves the value of switching essentially unchanged. The ANN produces diagonal arrows instead, indicating that additional attended evidence simultaneously decreases the value of staying and increases the value of switching. This coordinated change means that although the overall probability of sampling versus selecting may change little, the relative probability of switching attention to the unattended patch rises. Thus the ANN has learned a cross-patch integration that the fixed-form baselines miss, foreshadowing the information-symmetry principle made explicit by the symbolic regression in the next section.

### The ANN can be transformed into an interpretable symbolic function

To obtain a quantitative interpretation of the ANN’s learned function, we turned to symbolic regression, a machine-learning method that searches the space of analytic expressions to find an interpretable closed-form equation that approximates a target function^39^. Symbolic regression is particularly useful for neural-network interpretation: it produces human-readable expressions^40^ and dramatically reduces the number of parameters (here 7,592 trainable ANN units) to a few interpretable ones.

**Fig. 5:**
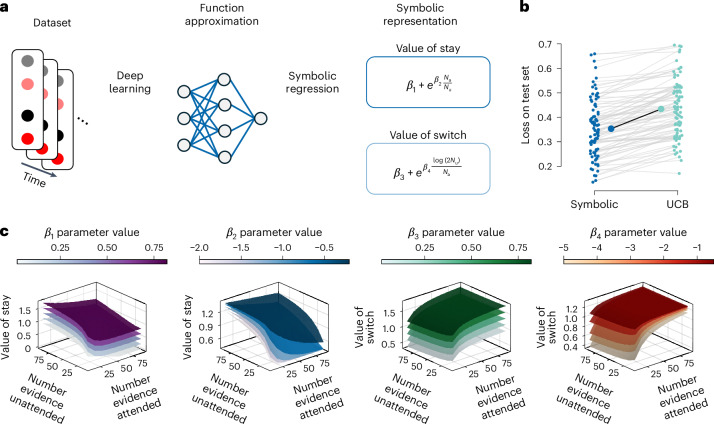
Symbolic representation of the ANN-derived VoI function. **a**, Process of deriving interpretable mathematical expressions from behavioral data. Left: schematic of the dataset containing patterns of dots across time. Middle: function approximation using a deep neural network. Right: symbolic representation showing the mathematical equations derived through symbolic regression for the value of staying and the value of switching, where *N*_a_ and *N*_u_ represent the number of dots in the attended and unattended patches, respectively. **b**, Cross-task generalization comparison. Scatter plot showing leave-one-out cross-validation loss on a test set from a two-armed bandit task^20^ for both the symbolic model (left) and the UCB model (right). Each gray line connects performance of both models for the same participant. Statistical comparison used a two-sided Wilcoxon signed-rank test on per-subject mean losses (*W* = 15.0, median difference = −0.07, *P* = 4.31 × 10^−16^). *n* = 89 participants. **c**, Parameter sensitivity analysis. Three-dimensional surface plots showing how the value functions change with different parameter values. Each surface represents the function with a specific parameter value according to the color scales above each plot. Source data

**Extended Data Fig. 6:**
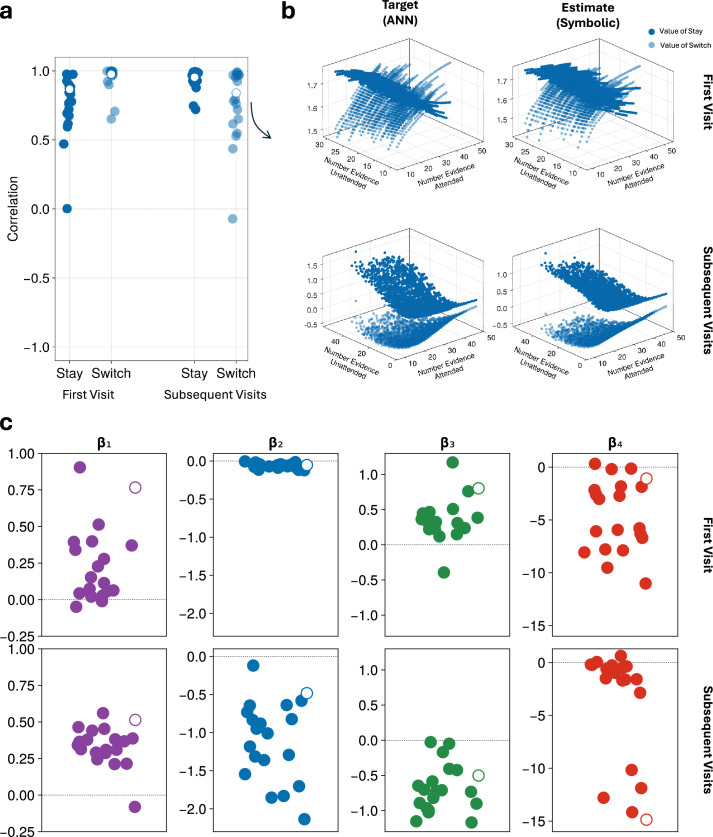
Symbolic regression approximation of ANN-derived value-of-information functions. **a**, Correlation between ANN-generated value of information and the symbolic-function approximations across all participants (n = 20). Four correlations are shown: value of staying and value of switching, each for first visits and for subsequent visits to a patch. Each point represents one participant; the highlighted participant (white circle) is shown in detail in panel b. **b**, Example participant showing 3D visualizations of the value-of-information functions. Left column: target functions from the trained ANN. Right column: estimates from the optimized symbolic functions. Top row: first-visit functions; bottom row: subsequent-visit functions. Dark points represent value of staying; light points represent value of switching. **c**, Individual parameter estimates across all participants. Each parameter corresponds to a specific component of the value-of-information computation: *β*_1_(1), *β*_1_( > 1) (stay-VoI offsets, ’attentional inertia’); *β*_2_(1), *β*_2_( > 1) (stay-VoI scaling, ’information satiation’); *β*_3_(1), *β*_3_( > 1) (switch-VoI offsets, ’undirected exploration’); *β*_4_(1), *β*_4_( > 1) (switch-VoI scaling, ’directed exploration’), for first (superscript 1) and subsequent (superscript >1) visits respectively. The highlighted participant (white circle) corresponds to the example shown in panel b. Source data

We extracted a mathematical expression for the value of stay and one for the value of switch. For the value of stay, we extracted the following expression ${\beta }_{1}+\exp (-| {\beta }_{2}| \,{N}_{{\rm{a}}}/{N}_{{\rm{u}}})$ (Fig. 5a), where *N*_a_ and *N*_u_ represent the number of dots revealed in the attended and unattended patches, respectively. For the value of switch, we found ${\beta }_{3}+\exp (-| {\beta }_{4}| \,\log (2{N}_{\mathrm{u}})/{N}_{{\rm{a}}})$ (Fig. 5a). These equations can be interpreted in terms of four psychological constructs. The parameter *β*_1_ reflects attentional inertia, the baseline tendency to continue sampling from the currently attended option (Fig. 5c, left). The parameter *β*_2_ captures information satiation, the rate at which accumulated evidence reduces the desire to continue sampling; notably, *β*_2_ is negative, meaning the value of staying decreases as *N*_a_/*N*_u_ increases (Fig. 5c, second from left). The parameter *β*_3_ reflects undirected exploration, the baseline tendency to explore alternatives regardless of evidence state. The parameter *β*_4_ captures directed exploration, the rate at which learning about the current option increases interest in alternatives; *β*_4_ is also negative, so the value of switching increases as *N*_a_ grows relative to *N*_u_ (Fig. 5c, right). Critically, information satiation (*β*_2_) and directed exploration (*β*_4_) work in concert: as evidence accumulates from the attended option, staying becomes less appealing while switching becomes more attractive, creating a coordinated transition from exploitation to exploration. This differs from UCB-style models. UCB exploration bonuses are linked to Bayesian posterior uncertainty: as samples accumulate, the posterior distribution over an option’s value narrows, reducing the exploration bonus^20^. UCB thus asks ‘how uncertain am I about this option?’ and computes a quantity based on absolute sample counts: that is, sampling an option 100 times versus 10 times yields very different bonuses. In contrast, *N*_a_/*N*_u_ asks ‘how does my knowledge of this option compare to the other?’ The ratio is scale invariant: whether 100:10 or 10:1, both yield *N*_a_/*N*_u_ = 10. This suggests that rather than computing posterior uncertainty to guide exploration, participants seek information symmetry: balancing knowledge across options rather than minimizing uncertainty about each independently. Notably, although these equations contain four core parameters, our feature selection analysis revealed that the visit number (first visit of the patch versus subsequent visits) was critical for optimal ANN performance. Consequently, each parameter is fitted independently for first visits versus subsequent visits, yielding eight total parameters that capture distinct sampling strategies across different phases of exploration. We denote first-visit parameters with superscript (1) (for example, ${\beta }_{1}^{(1)}$) and subsequent-visit parameters with superscript (>1) (for example, ${\beta }_{1}^{( > 1)}$). These interpretable parameters provide insight into individual differences in sampling strategies (Extended Data Fig. 6c).

To examine whether these symbolic functions accurately capture the function learned by the ANN, we replaced the ANN component in our hybrid model (Fig. 3a, bottom) with these newly discovered functions and assessed how well this variant explained participant behavior. We found that the symbolic-hybrid model and ANN-hybrid had comparable power to explain participant behavior (Fig. 3b), suggesting that we had successfully discovered a transparent and interpretable mathematical relationship between evidence and the VoI.

Finally (Fig. 5b), we tested whether these symbolic functions capture general principles of information sampling rather than task-specific features. We evaluated performance of the symbolic model on an independent dataset where participants completed a two-armed bandit task^20^. Participants in this study performed a very different task from ours (for example, they repeatedly chose between two options and received point rewards), yet in both tasks participants had to strike a balance between exploration and exploitation to maximize their rewards. Both UCB and symbolic models use the sample count *N* to compute exploration bonuses. In our task, *N* represents the number of dots sampled from a patch, whereas in the bandit task, *N* represents the number of times an arm was chosen. This makes *N* the natural mapping variable between tasks. Remarkably, our symbolic functions also outperformed the UCB model in predicting participants’ choices in this different context (Wilcoxon signed-rank test: *W* = 15.0, *n* = 89, median difference = −0.07, *P* = 4.31 × 10^−16^). We noticed that one simple difference between the standard UCB formulation and our symbolic model is that the UCB lacks an offset parameter, whereas our symbolic model includes one. To ensure that this superior performance was not simply due to the model’s ability to adjust the baseline offset, but rather reflected a fundamental difference in the shape of the VoI function, we tested an UCB model that included an offset parameter in its exploration bonus computation. Even when compared to this more flexible UCB variant, the symbolic model still demonstrated significantly better predictive performance (Wilcoxon signed-rank test: *W* = 286.0, *n* = 89, median difference = −0.03, *P* = 2.21 × 10^−12^). These results suggest that the equations discovered with symbolic regression capture fundamental aspects of human exploration behavior that are not specific to the information-sampling task we used and cannot be accounted for by simple parametric extensions to existing models.

### The ANN-derived VoI can predict neural activity

Next, we examined whether the ANN-derived VoI could also predict neural activity. Participants performed the task while undergoing ultrahigh-field 7T fMRI with 1-mm isotropic voxels, using a limited field of view that captured key regions of interest (ROIs) in the midbrain, brainstem and interconnected cortical areas (Supplementary Fig. 1). We employed a general linear model (GLM) using VoI as a parametric modulator, controlling for outcome and VoS, and compared fit across four VoI computations (linear, UCB, ANN-derived and symbolic) using per-voxel mean squared error (MSE) between observed and model-predicted blood-oxygenation-level-dependent (BOLD) time series (Methods). Note that (unlike for behavior in Fig. 3b) this comparison fits the same number of parameters per model, using each model’s VoI output as a parametric modulator.

**Extended Data Fig. 7:**
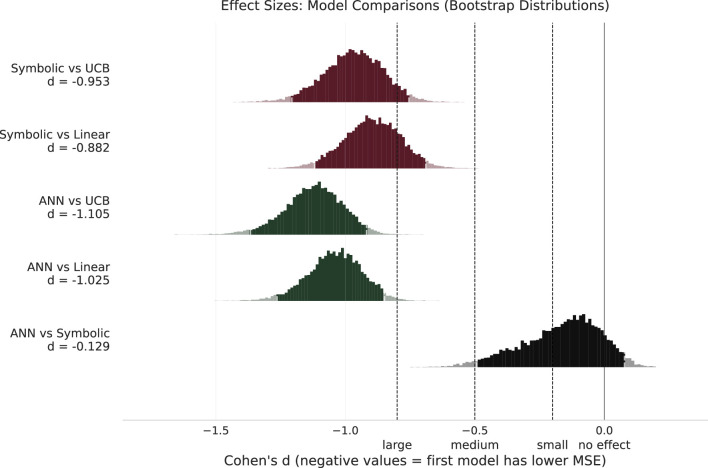
Neural model comparison across whole brain. Ridge plots showing the bootstrap distributions of Cohen’s d effect sizes for pairwise model comparisons in predicting neural activity (n = 80 sessions, two-sided Wilcoxon signed-rank tests). The ANN vs. Symbolic comparison (bottom, black) shows a small effect size (d = − 0.129, 95% CI [ − 0.494, 0.078], Wilcoxon P = 0.006), with a confidence interval overlapping zero, indicating equivalent performance. In contrast, ANN vs. Linear (dark green, d = − 1.025, 95% CI [ − 1.264, − 0.849], Wilcoxon P = 2.12 × 10^−12^) and ANN vs. UCB (dark green, *d* = − 1.105, 95% CI [ − 1.366, − 0.918], Wilcoxon P = 5.64 × 10^−13^) show large negative effects, indicating substantially better ANN performance. Symbolic vs. Linear (dark red, d = − 0.882) and Symbolic vs. UCB (dark red, d = − 0.953) also show large effects, demonstrating that the symbolic function outperforms standard heuristics while matching ANN performance. Darker shaded regions within each distribution represent 95% confidence intervals. Vertical reference lines indicate conventional effect-size thresholds: no effect (0), small (0.2), medium (0.5), and large (0.8). Negative Cohen’s d values indicate the first model has lower MSE (better fit). Source data

Consistent with our behavioral findings, the ANN-derived VoI provided a better overall fit to neural data across the brain. To quantify these differences, we used Cohen’s *d* effect sizes with bootstrap confidence intervals, complemented by Wilcoxon signed-rank tests (Extended Data Fig. 7): effect sizes quantify the magnitude of differences, whereas significance tests detect consistent directional effects regardless of size. The ANN-derived VoI outperformed both linear (Cohen’s *d* = − 1.025, Wilcoxon *P* = 2.12 × 10^−12^) and UCB models (Cohen’s *d* = − 1.105, Wilcoxon *P* = 5.64 × 10^−13^) with large effect sizes. Critically, the interpretable symbolic function performed almost as well as the original ANN (Cohen’s *d* = − 0.129, Wilcoxon *P* = 0.006); the negligible effect size (well below the ∣0.2∣ threshold) indicates that the symbolic abstraction substantially preserves the ANN’s predictive accuracy while providing mechanistic insight. The computational principles captured by our hybrid ANN therefore not only better describe participants’ behavior but also more accurately reflect the underlying neural computations. We then investigated activity in specific brain regions.

### AI and ACC covary with the ANN-derived VoI

To identify the neural correlates of the ANN-derived VoI, we conducted two complementary analyses. First, we performed a whole-brain GLM analysis to explore cortical regions most strongly associated with the ANN-derived VoI. Second, we conducted targeted analyses on predefined ROIs at the origins of the neuromodulatory systems, DRN, LC, VSN, VTA and SN (Fig. 1d). The neuromodulators associated with each of these nuclei have been proposed previously as mediators of the impact of uncertainty on decision-making^3,21–27^. In general, unlike the cortical regions, they are too small to survive cluster correction analysis strategies that incorporate a thresholding criterion based on the spatial extent of the activity^28,41,42^.

**Fig. 6:**
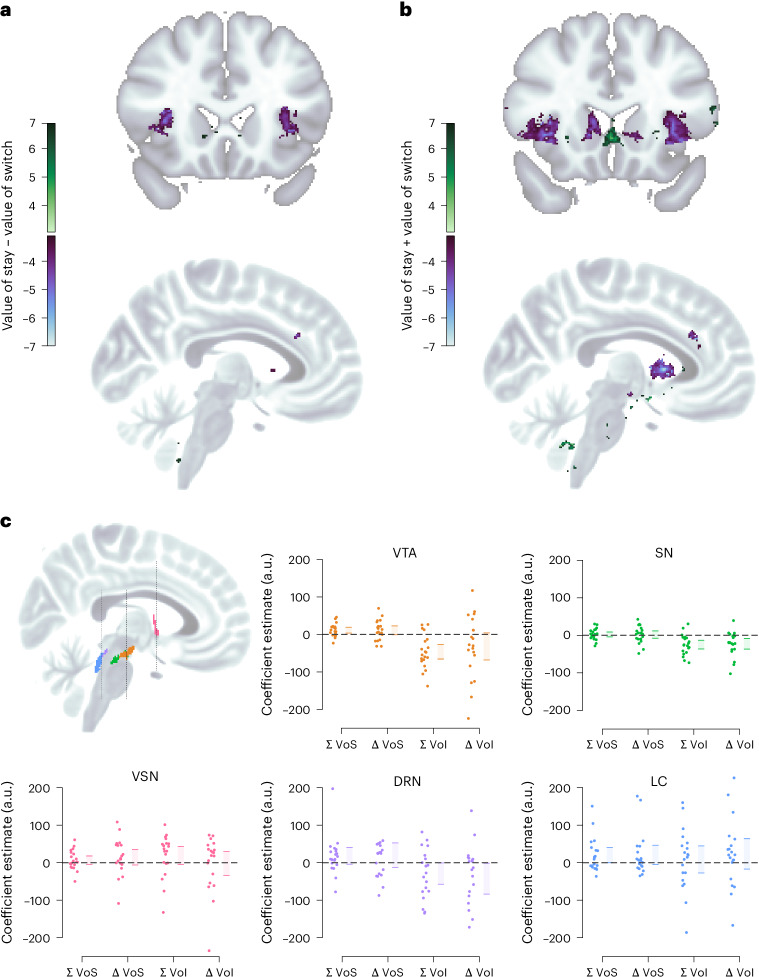
Neural correlates of ANN-derived value signals. **a**, Whole-brain analysis showing regions where BOLD activity correlates with the difference between the value of staying and the value of switching (VoI stay − VoI switch). **b**, Whole-brain analysis showing regions correlating with the sum of the value of staying and switching (VoI stay + VoI switch). Color bars indicate *Z*-statistic values; thresholded at *Z* > 3.1, cluster-corrected *P* < 0.001. **c**, ROI analysis results for neuromodulatory nuclei. Left: sagittal view showing locations of VTA (orange), SN (green), VSN (pink), DRN (purple) and LC (blue). Right: coefficient estimates (effect sizes from weighted mixed-effects models on beta values) for regressors representing the sum (Σ) and difference (Δ) of the VoS and VoI within each ROI. Each small dot represents an individual participant’s random-effect estimate plus the fixed effect; shaded bars indicate the 95% confidence interval of the fixed effect (group mean). *n* = 20 participants (×4 sessions per participant). Source data

The whole-brain analysis revealed that activity in the AI and ACC was associated with the ANN-derived VoI (Fig. 6a,b). AI activity exhibited a negative association with the difference between value of stay and value of switch (Fig. 6a, top) and a negative association with the sum of value of stay and value of switch (Fig. 6b, top); in other words, AI activity increased as the value of switching attention increased and decreased as the value of maintaining attention at the current location increased. The ACC showed the same pattern (Fig. 6b). These computational signatures appear crucial for guiding participants’ decisions about whether to stay with the current patch or switch to the alternative (Fig. 1b, bottom) and whether to keep sampling information or stop the learning process and initiate an option selection (Fig. 1a). Model comparisons within AI and ACC ROIs confirmed that the hybrid-ANN and symbolic models substantially outperformed linear and UCB, with no significant difference between ANN and symbolic (Supplementary Table 1).

Analysis of the subcortical ROIs revealed a distinct pattern in the VTA (Fig. 6c), which showed positive coding of the sum of VoS (*β* = 11.3, *P* = 0.020) but negative coding of the sum of VoI (*β* = −46.1, *P* = 1.93 × 10^−5^). This activity pattern would be sufficient to guide participants’ decisions about whether to continue sampling information from the patch they are attending or to make a final selection (Fig. 1a, bottom right versus top right).

The SN exhibited a pattern similar to AI and ACC (Fig. 6c): its activity was negatively associated with both the sum (*β* = −24.9, *P* = 6.38 × 10^−5^) and the difference (*β* = −22.7, *P* = 0.008) of the VoI, suggesting that SN tracks both the overall potential for information gain and the relative informational value of the current versus alternative option.

There has been particular interest in the possibility that the LC, and the noradrenergic system with which it is linked, the DRN, and its serotonergic system, or the VSN, associated with the cholinergic system, encode uncertainty. Our high-field fMRI recordings gave us a unique opportunity to test these hypotheses in humans. We first examined whether LC, DRN or VSN activity can predict the ANN-derived VoI, an index closely, but inversely, related to uncertainty.

Within the anatomically defined VSN, DRN and LC ROIs (Fig. 6c), we found no significant association between BOLD activity and the sum or difference of the VoI or VoS (all *P* values Bonferroni-corrected across ROIs). Within the sensitivity limits of our measurement and analysis, these specific neuromodulatory nuclei do not strongly encode these decision variables in this task.

**Extended Data Fig. 8:**
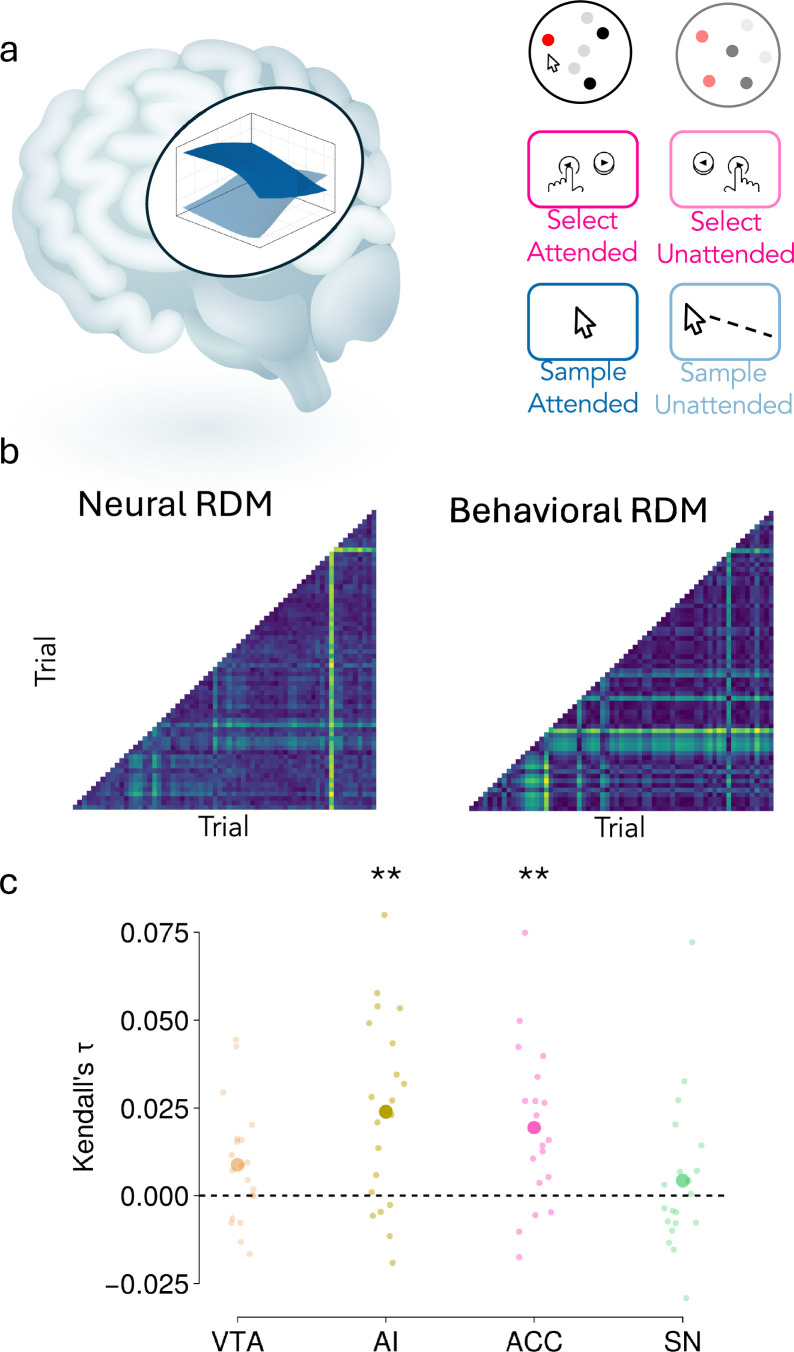
Representational Similarity Analysis links neural patterns to sampling behaviour. **a**, Schematic illustrating the relationship between brain activity and behavior. **b**, Example Neural RDM (left, based on multi-voxel BOLD activity patterns) and Behavioral RDM (right, based on the number of samples taken per trial) for a participant session. Each cell represents the dissimilarity between a pair of trials; higher values (yellow) indicate greater dissimilarity. **c**, Kendall’s *τ* correlation coefficients quantifying the relationship between neural and behavioral RDMs for VTA, AI, ACC, and SN. Neural RDMs for AI, ACC, and SN were derived using masks matched in shape and size to the VTA ROI to control for region size. Each small dot represents the correlation calculated for one participant session; larger dots indicate the mean correlation across participants. Asterisks denote significant positive correlations across participants (two-sided Wilcoxon signed-rank test; ** P < 0.01, Bonferroni corrected). AI: *τ* = 0.024, P = 0.0006, Bonferroni corrected P = 0.0024; ACC: *τ* = 0.019, P = 0.0004, Bonferroni corrected P = 0.0017; VTA: *τ* = 0.008, P = 0.072; SN: *τ* = 0.004, P = 0.46, Bonferroni corrected P = 1.0. n = 20 participants ( × 4 sessions per participant). Source data

To comprehensively assess model performance across subcortical regions, we compared the MSE between models within all five subcortical ROIs (VTA, SN, DRN, LC, VSN). First-level GLMs fitted using ANN-derived VoI demonstrated significantly lower MSE than those fitted using linear-derived VoI across all subcortical regions and significantly lower MSE than those fitted using UCB-derived VoI in four of the five regions (all except LC after Bonferroni correction; Supplementary Table 1). This consistent superiority of the ANN-derived approach across diverse neuromodulatory nuclei further supports the conclusion that the computational principles captured by our hybrid model better reflect the underlying neural mechanisms of information valuation, even in regions where the VoI signal itself may not reach statistical significance. Multivariate representational similarity analysis further confirmed that activity patterns in AI and ACC, but not in VTA or SN, covaried with trial-by-trial sampling duration (Extended Data Fig. 8 and Supplementary Note 1).

## Discussion

Information seeking has been proposed to be a fundamental behavior in humans and other animals^3,21,43,44^. Understanding how and why information is valued bears on how people interact with information sources from other individuals to the internet^45,46^, how information seeking can become pathological or maladaptive^47,48^ and how people and animals resolve the fundamental trade-off between gathering evidence and committing to a decision. In the current study, we examined how the VoI is itself determined.

Four key parameters (Fig. 5a,c) determine VoI obtained from the current focus of attention as opposed to VoI at an alternative location. The first and third, *β*_1_ (attentional inertia) and *β*_3_ (undirected exploration), reflect baseline tendencies to continue sampling the current option or to explore alternatives, respectively. The second and fourth, *β*_2_ (information satiation) and *β*_4_ (directed exploration), capture how accumulated evidence shapes these tendencies: as participants learn more about the attended option, the value of staying decreases and the value of switching increases. This coordinated pattern means that information-seekers do not exhaustively explore one option before considering alternatives; instead, they balance knowledge across available choices, shifting attention toward less explored options as information accumulates. Notably, the parameter estimates differ between first and subsequent visits to a patch (Extended Data Fig. 6c). During first visits, the information satiation parameter has a smaller magnitude ($| {\beta }_{2}^{( > 1)}| > | {\beta }_{2}^{(1)}|$), suggesting that participants gather a minimum amount of information before switching rather than dynamically adjusting based on relative evidence. The undirected exploration parameter is lower during subsequent visits (${\beta }_{3}^{( > 1)} < {\beta }_{3}^{(1)}$), reflecting reduced exploration drive or an increased effective cost of switching. This pattern is adaptive: during first visits, participants must switch to learn the red–black distribution of the unattended patch, justifying the time cost of switching. During subsequent visits, participants already have an estimate of the number of red and black dots from previous visits, so switching offers less informational benefit while incurring the same time cost. A lesion analysis of the symbolic equations confirmed that the logarithmic transformation in the value-of-switching equation captures an essential nonlinearity: diminishing sensitivity to evidence about the unattended option (Supplementary Note 2 and Supplementary Fig. 2).

The flexible ANN approach captured nonlinear relationships between choice features and VoI without requiring a prior hypothesis about their form. Symbolic regression then rendered the learned relationship interpretable without sacrificing neural prediction accuracy. The symbolic function predicted whole-brain and region-specific neural activity with precision comparable to the original ANN, demonstrating that the abstraction captures the computationally relevant features of the learned VoI function.

An alternative approach would be to train an end-to-end ANN that maps task states directly to action probabilities without imposing cognitive structure. Although such an unconstrained model is very expressive, it is harder to interpret. The hybrid approach constrains the ANN to learn only a subcomponent of the decision process (the mapping from evidence counts to VoI), rather than the full state-to-action mapping. This constraint reduces the risk that different internal representations yield equivalent predictions: the cognitive scaffold specifies the role that the ANN’s output must play (VoI), narrowing the space of functions that can achieve good predictions. In contrast, an unconstrained end-to-end ANN could organize its internal computations in many different ways to achieve the same behavioral output. Additionally, the simpler function learned by the constrained ANN is more amenable to symbolic regression. Crucially, the hybrid approach produces psychologically interpretable intermediate variables, such as VoI, that can serve as regressors in neuroimaging analyses, enabling the brain–behavior linking that is central to cognitive neuroscience.

The model has some generalizability: it predicted behavior in a different explore–exploit task^20^ (Fig. 5b). Two limitations remain. First, the specific equations are tailored to our two-option structure, encoding relative comparisons between attended and unattended options; unlike UCB, they do not naturally extend to *n*-armed bandits. Possible extensions include replacing *N*_unattended_ with the mean across unattended options or weighting unattended options by factors such as salience or recency. Second, our task does not permit dissociation of decision uncertainty (‘which option is better?’), evidence uncertainty (‘how much evidence have I collected?’) and reward uncertainty (‘what outcome will I receive?’), which are highly correlated in our design; orthogonal manipulation of these uncertainty types is an important direction for future research.

There is a growing recognition that purely theory-driven models may sometimes oversimplify complex cognitive processes, and purely data-driven approaches like deep learning often suffer from a lack of interpretability. Recent work explores various ways to bridge this gap, often falling into two main streams. One stream focuses on leveraging neural networks while enhancing interpretability: this includes explicitly integrating ANNs within classic cognitive frameworks^38^ or utilizing deep learning architectures specifically designed for interpretability or cognitive plausibility, such as tiny^16^ or disentangled^49^ recurrent neural networks. A second, less explored, stream aims for interpretability by discovering mathematical equations directly from behavioral data using equation-discovery algorithms^50–52^. The workflow we propose integrates aspects of both these streams. First, we use a flexible ANN to learn complex input–output mappings without strong a priori constraints. Then, we use symbolic regression to distill these learned mappings into human-readable equations. A natural question that arises is, why not apply symbolic regression directly to behavioral data rather than first fitting an ANN? There are several computational and methodological reasons for our two-stage approach. First, direct symbolic regression on the complex mapping from task state to behavior would involve an intractably large search space. By first learning this mapping with ANNs and then applying symbolic regression to the learned function, we factorize this complex problem into manageable components, dramatically reducing the search space from multiplicative to additive complexity. Second, our neural-network architecture (Lipschitz-bounded deep network^53^) inherently enforces smoothness constraints on the learned function, providing stability that would not be guaranteed with direct symbolic regression. Third, and crucially, our symbolic regression operates within a broader cognitive model optimized via gradient descent. Embedding a nondifferentiable genetic algorithm (symbolic regression) directly within this gradient-based optimization framework would create substantial computational challenges. By replacing the VoI computation with a differentiable neural network, we transform this into a standard, tractable optimization problem. This approach thus combines the computational efficiency and stability of neural-network training with the interpretability benefits of symbolic regression. This approach was central to the current study but is likely to have wider applicability in cognitive science and neuroscience. We believe it holds considerable potential for refining existing theories and discovering computational principles across diverse domains, from learning and decision-making to perception and social cognition.

All major neuromodulatory systems have at one time or another been linked to uncertainty processing^3,21–27^, but identifying a specific or preeminent contribution has been difficult. Recording simultaneously from VTA, SN, DRN, LC and VSN with 7T fMRI, we found VTA activity showed opposed coding of the sum of VoI and the sum of VoS (Fig. 6c), a pattern suited to arbitrating between continued sampling and final choice. SN exhibited a related pattern plus sensitivity to the difference in information value between options, consistent with encoding both overall potential for information gain and the relative value of the current versus alternative option. Similar patterns emerged in ACC and AI (Fig. 6a,b), which project to or are adjacent to VTA and SN^3,22,29–33,54,55^ (with multisynaptic routes via habenula^56,57^). Crucially, multivariate activity patterns in ACC and AI, but not in VTA or SN, covaried with trial-by-trial sampling duration (Extended Data Fig. 8 and Supplementary Note 1), indicating that these cortical regions are especially involved in information evaluation.

Previous studies have identified ACC and VTA and anatomical structures that interconnect them, such as habenula, in evaluation of information. Activity in individual neurons in both habenula and VTA reflects both the value of a stimulus in terms of the reward that it predicts^58–60^ as well as VoI^21,22,27,61^, in line with the suggestion that VTA may compare the relative advantage to be gained from making a selection and seeking more information about a choice. Activity in ACC has also been linked to information seeking and initiation of behavioral change^14,17,22,62–66^, consistent with the proposal that ACC might arbitrate when it is advantageous to seek more information about a current opportunity and when it is better to pursue an alternative. AI is relatively less investigated, but in line with the current findings, AI and activity in dopaminergic midbrain areas such as SN have been reported when people evaluate whether and when to initiate an action^28,41^.

## Methods

### Subjects

Twenty participants (14 females), aged 19 to 32 years, completed the study. All participants were paid £15 per hour and an additional performance-dependent bonus of between £20 to £40 for rewards collected during the task. Each participant provided written informed consent at the beginning of each testing session. Ethical approval was given by the Oxford University Central University Research Ethics Committee (Ethics Approval Reference: R82877/RE001). Behavioral data from all participants were used for the analysis.

### Experimental task

On each trial, participants were shown three patches of 100 moving dots each. Each dot’s true color was red or black, but dots were initially hidden under green or gray covers. Green covers were revealed simultaneously upon a patch’s first visit; gray covers were revealed sequentially (one dot every 150 ms) while a participant hovered over the patch. The number of gray-covered dots per patch varied trial by trial between 70 and 95, dissociating sampling duration from initial uncertainty. The trial sequence was as follows: (1) participants clicked a start button; (2) they visited each patch to reveal its green-covered dots; (3) they clicked a central sampling button to begin the information-gathering phase; (4) on 80% of trials, one of the three patches was randomly blocked and could not be sampled or chosen—the remaining 20% of three-option trials ensured attention to all initial uncertainties and strengthened the two-option trials used in all subsequent analyses; (5) participants freely sampled by hovering over patches, with gray-covered dots revealed sequentially. Revealed colors were visible only while a patch was attended; switching away returned its revealed dots to the gray-covered state, forcing participants to rely on memory. When returning to a previously visited patch, previously revealed dots remained revealed, and any still gray-covered dots continued to reveal sequentially. Sampling was capped at 24 s for two-option trials and 33 s for three-option trials. Participants then chose a patch using an MRI-compatible button-box and received feedback via a ‘duel’ animation (1,300 ms) and a win/lose image (1,500 ms). Each participant completed four 25-min sessions, incentivized with a £5 bonus per session for maximizing correct choices. The experiment was implemented in jsPsych (v6.3.0). To increase engagement, the task was framed as a medieval game (‘The Last Duel’) in which participants chose a weapon to face an opponent armed with a hammer. Each patch represented a weapon store: the patch with the highest proportion of red dots contained a sword (100% win rate), the intermediate proportion a hammer (50%) and the lowest a stone (0%).

### Behavioral analysis

We used mixed-effects logistic regression to characterize three aspects of choice behavior. First, we modeled the probability of staying at versus switching from the currently attended patch (Fig. 1a, bottom):

1 $$\begin{array}{ll}{\mathrm{logit}}({\mathrm{stay}}_{i,\,j}) & =({\beta }_{0}+{u}_{0,\,j})+({\beta }_{1}+{u}_{1,\,j})\times {\mathrm{{VoI}}}_{{\rm{a}}}+({\beta }_{2}+{u}_{2,\,j})\times {\mathrm{VoI}}_{{\rm{u}}}\\ & +({\beta }_{3}+{u}_{3,\,j})\times {\mathrm{first}}\,\_{\mathrm{visit}}+({\beta }_{4}+{u}_{4,\,j})\times {\mathrm{VoI}}_{{\rm{a}}}\times {\mathrm{first}}\_\,{\mathrm{visit}}\\ & +({\beta }_{5}+{u}_{5,\,j})\times {\mathrm{VoI}}_{{\rm{u}}}\times {{\mathrm{first}\_{\mathrm{visit}}}}+({\beta }_{6}+{u}_{6,\,j})\times ({\mu }_{{\rm{a}}}-{\mu }_{{\rm{u}}})\\ & +({\beta }_{7}+{u}_{7,\,j})\times {({\mu }_{{\rm{a}}}-{\mu }_{{\rm{u}}})}^{2}\end{array}$$

Second, we modeled the probability of continuing to sample versus making a final selection, using the absolute difference and sum of the VoI, their interactions with first-visit indicator,and the absolute difference and sum of the observed red-dot proportions:

2 $$\begin{array}{ll}\mathrm{logit}({\mathrm{sample}}_{i,j}) & =({\beta }_{0}+{u}_{0,j})+({\beta }_{1}+{u}_{1,j})\times |{\mathrm{VoI}}_{{\rm{a}}}-{\mathrm{VoI}}_{{\rm{u}}}|\\ & +({\beta }_{2}+{u}_{2,j})\times ({\mathrm{VoI}}_{{\rm{a}}}+{\mathrm{VoI}}_{{\rm{u}}})+({\beta }_{3}+{u}_{3,j})\times \mathrm{first}\,\_{\mathrm{visit}}\\ & +({\beta }_{4}+{u}_{4,j})\times |{\mathrm{VoI}}_{{\rm{a}}}-{\mathrm{VoI}}_{{\rm{u}}}|\times {\mathrm{first}}\_\,{\mathrm{visit}}\\ & +({\beta }_{5}+{u}_{5,j})\times ({\mathrm{VoI}}_{{\rm{a}}}+{\mathrm{VoI}}_{{\rm{u}}})\times {\mathrm{first}}\_{\mathrm{visit}}+({\beta }_{6}+{u}_{6,j})\times \\ & |{\mathrm{mean}}_{{\rm{a}}}-{\mathrm{mean}}_{{\rm{u}}}|+({\beta }_{7}+{u}_{7,j})\times ({\mathrm{mean}}_{{\rm{a}}}+{\mathrm{mean}}_{{\rm{u}}})\end{array}$$

Third, we modeled the final patch selection as a function of the observed means and the VoI in both patches:

3 $$\begin{array}{lll}{\rm{logit}}({\rm{select}}\,{\rm{attended}}) & = & ({\beta }_{0}+{u}_{0,\,j})+({\beta }_{1}+{u}_{1,\,j})\times {{\rm{mean}}}_{{\rm{a}}}\\ & & +({\beta }_{2}+{u}_{2,\,j})\times {{\rm{mean}}}_{{\rm{u}}}\\ & & +({\beta }_{3}+{u}_{3,\,j})\times {{\rm{VoI}}}_{{\rm{a}}}\\ & & +({\beta }_{4}+{u}_{4,\,j})\times {{\rm{VoI}}}_{{\rm{u}}}\end{array}$$

Here *i* indexes observations, *j* indexes subjects, *β*_*k*_ are fixed effects, *u*_*k*, *j*_ are subject-specific random effects with ${{\bf{u}}}_{j} \sim {\mathcal{N}}(0,\Sigma )$; VoI_a_, VoI_u_ are the VoI for the attended and unattended patches and *μ*_a_, *μ*_u_ are the observed proportions of red dots in each. Each model was fitted three times, once for each VoI function (linear, UCB, ANN), using MixedModels.jl in Julia.

### Markov decision process–based model

We computed a reference policy by solving the task as a Markov decision process using backward induction. This policy is ‘optimal’ in the sense that it maximizes expected reward given the model’s specific cost assumptions; it is not intended to represent uniquely correct human behavior. Full formalization (state/action space, beta–binomial transitions, reward function and Bellman recursion) is given in Supplementary Note 3.

### Hybrid model

To model the choice data, we used a hybrid model that computes action values for four actions: sampling from or selecting either of the two available patches. The model takes as input the number of revealed dots (red + black) in both patches, the number of red dots in both patches and the number of green dots in the blocked patch and transforms these objective quantities into subjective magnitudes in a visit-dependent way. On the first visit to a patch, the model accounts for interference from the blocked patch when estimating the unattended patch’s contents:

4 $$\widehat{{n}_{{\rm{u}}}}=\omega {n}_{{\rm{u}}}+(1-\omega ){n}_{{\rm{b}}}$$

where *n*_u_ is the actual number of green dots in the unattended patch and *n*_b_ in the blocked patch and *ω* ∈ [0, 1] quantifies resistance to interference (*ω* = 1: no interference). The unattended patch’s red dots are anticipated from the attended patch’s red proportion:

5 $$\widehat{{r}_{{\rm{u}}}}=\widehat{{n}_{{\rm{u}}}}(0.5+(\widehat{{\mu }_{{\rm{a}}}}-0.5){\lambda }_{0})$$

6 $$\widehat{{\mu }_{{\rm{a}}}}=\frac{\widehat{{r}_{{\rm{a}}}}}{\widehat{{n}_{{\rm{a}}}}}$$

where *λ*_0_ is a free parameter that controls how strongly the proportion of red dots in the attended patch ($\widehat{{\mu }_{{\rm{a}}}}$) influences expectations about the unattended patch.

On subsequent visits, revealed dots in the unattended patch return to their gray-covered state when participants switch away, so the model represents the unattended patch via exponentially decaying memory:

7 $$\widehat{{n}_{{\rm{u}}}}={n}_{{\rm{u}}}{{\rm{e}}}^{-{\lambda }_{2}t}$$

8 $$\widehat{{r}_{{\rm{u}}}}={r}_{{\rm{u}}}{{\rm{e}}}^{-{\lambda }_{2}t}$$

where *t* is time elapsed since the current visit began and *λ*_2_ is the decay rate. Because $\widehat{{n}_{{\rm{u}}}}$ and $\widehat{{r}_{{\rm{u}}}}$ decay at the same rate, uncertainty about the unattended red proportion grows over time. For the attended patch, subjective magnitudes equal the objective ones.

From these subjective magnitudes, the value of selecting each patch is the difference in estimated red proportions:

9 $${Q}_{\mathrm{select}\,\mathrm{attended}}=\widehat{{\mu }_{{\rm{a}}}}-\widehat{{\mu }_{{\rm{u}}}}$$

10 $${Q}_{\mathrm{select}\,\mathrm{unattended}}=\widehat{{\mu }_{{\rm{u}}}}-\widehat{{\mu }_{{\rm{a}}}}$$

11 $$\widehat{{\mu }_{{\rm{u}}}}=\frac{\widehat{{r}_{{\rm{u}}}}}{\widehat{{n}_{{\rm{u}}}}}$$

and the value of continuing to sample depends on whether the participant stays or switches:

12 $${Q}_{\mathrm{sample}\,\mathrm{attended}}=\mathrm{value}\,\_\mathrm{of}\_\,\mathrm{stay}(\widehat{{n}_{{\rm{a}}}},\widehat{{n}_{{\rm{u}}}})$$

13 $${Q}_{\mathrm{sample}\,\mathrm{unattended}}=\mathrm{value}\,\_\mathrm{of}\_\,\mathrm{switch}(\widehat{{n}_{{\rm{a}}}},\widehat{{n}_{{\rm{u}}}})-\kappa$$

where *κ* captures the cognitive cost of switching attention. We implemented and compared three approaches for the value_of_stay and value_of_switch functions: linear, UCB and ANN. The linear function is

14 $$\mathrm{linear}(n)={\beta }_{1}+n\times {\beta }_{2}$$

where *β*_1_ is a baseline and *β*_2_ scales with the number of revealed dots. Although the linear function is computed per patch, cross-patch comparison emerges via the softmax: *P*(stay) depends on the difference linear(*N*_u_) − linear(*N*_a_) + *κ*. The UCB function is

15 $$\mathrm{ucb}(n,N)=\sqrt{\frac{\log (N)}{n}}\times \beta$$

where *N* is the total dot count across patches, *n* is the count in the current patch and *β* scales the exploration bonus. The ANN is a fully connected Lipschitz-bounded deep network with four hidden layers of 32 neurons each and tanh activations. The Lipschitz constraint guarantees that small input changes produce proportionally small outputs, yielding smooth value estimates. Inputs are the normalized dot counts $\widehat{{n}_{\mathrm{left}}}/100$ and $\widehat{{n}_{\mathrm{right}}}/100$, binary cursor indicators and a first-visit flag; outputs are per-patch VoIs.

The ANN-based hybrid model was trained in three stages: (1) joint Adam optimization of the network and cognitive-module parameters, with learning rates *η*_nn_ = 0.01 for the neural network and *η*_cm_ = 0.02 for the cognitive model; (2) limited-memory Broyden–Fletcher–Goldfarb–Shanno (L-BFGS) refinement of cognitive parameters with the network fixed; (3) joint Adam fine-tuning with reduced rates (*η*_*nn*_ = 0.001, *η*_*cm*_ = 0.0001). The linear and UCB variants were fitted end-to-end with L-BFGS, minimizing cross-entropy loss between predicted and actual choices.

### Symbolic regression

To discover interpretable equations describing how participants compute the VoI, we ran symbolic regression (SymbolicRegression.jl^39^) on the hybrid ANN’s predictions. For each unique combination of attended (*N*_a_) and unattended (*N*_u_) dot counts (binned into 24 equally spaced bins each from 0 to 100), we averaged the ANN’s predicted values of staying and switching across the 20 participant-specific networks and then searched for closed-form expressions approximating these predictions. The search used the operators $\{+,-,\times ,/,\log ,\exp ,\sigma \}$, with exp, sigmoid and log each nestable at most once within their own type; the complexity of constants was set to 2 and the maximum expression size to 20 terms. Optimization ran for 10,000 iterations and selected equations by a combined score of prediction loss and expression complexity. Symbolic regression discovered

16 $$\,\mathrm{Value\; of\; staying}\,={\beta }_{1}+\exp \left(-| {\beta }_{2}| \frac{{N}_{\mathrm{attended}}}{{N}_{\mathrm{unattended}}}\right)$$

17 $$\,\mathrm{Value\; of\; switching}\,={\beta }_{3}+\exp \left(-| {\beta }_{4}| \frac{\log (2\times {N}_{\mathrm{unattended}})}{{N}_{\mathrm{attended}}}\right)$$

Because feature selection indicated that the first-visit indicator was critical (Supplementary Note 6), we fitted separate parameter sets for first versus subsequent visits (superscripts (1) and (>1) in Supplementary Table 2). During fitting, the switch-function offset *β*_3_ and the switch-cost parameter *κ*_1_ were both additive offsets to the switching value, making them nonidentifiable; we resolved this by absorbing their combined effect into the switch cost.

### Model-validation analyses

We performed four model-validation analyses whose full procedures are reported in the Supplementary Information: an end-to-end ANN benchmark establishing an upper bound on predictive performance (Supplementary Note 4), a parameter-recovery simulation confirming that the hybrid approach recovers known VoI functions (Supplementary Note 5), a leave-one-out feature-selection analysis identifying the task inputs critical for model performance (Supplementary Note 6) and a cross-task generalization test of the symbolic and UCB models on two external two-armed bandit datasets (Supplementary Note 7).

### Imaging data acquisition

Structural and functional MRI data were collected with a Siemens 7T MRI scanner. High-resolution functional data were acquired with a multiband gradient echo T2* echo planar imaging sequence with 1-mm isotropic voxels, multiband acceleration factor 2, repetition time (TR) = 1.378 s, echo time (TE) = 27 ms, flip angle = 90^∘^ and generalized autocalibrating partially parallel acquisition acceleration factor 2. The parameters were selected to maximize signal-to-noise ratio in subcortical areas. To accommodate the high temporal and spatial resolution of the protocol, functional scans had a limited field of view (FOV) oriented at 45 degrees with respect to the anterior commissure-posterior commissure line (36 slices). The FOV captured all ROIs in the midbrain, brainstem and cortex. Before acquiring the task-related functional scan, we acquired a presaturation single-measurement, whole-brain functional scan with the same orientation. The presaturation scan was used to facilitate registration of the limited-FOV task-related functional scan to the whole brain. Structural data were acquired using a T1-weighted magnetization prepared–rapid gradient echo sequence with 0.7-mm isotropic voxels, generalized autocalibrating partially parallel acquisition acceleration factor 2, TR = 2,200 ms, TE = 3.02 ms and inversion time = 1,050 ms. To correct distortions arising from inhomogeneities in the magnetic field, a fieldmap sequence was acquired with 2-mm isotropic voxels, TR = 620 ms, TE1 = 4.08 ms and TE2 = 5.1 ms. To account for the effects of physiological noise on functional MRI data, participants were fitted with a pulse oximeter and respiratory bellows that acquired cardiac and respiratory time series at 50 Hz using a BioPac MP160 device (BIOPAC Systems Inc.).

### fMRI data preprocessing

Preprocessing of fMRI data was performed with the FMRIB Software Library^67,68^. The Brain Extraction Tool^69^ was used to separate brain from nonbrain matter in structural and functional images. Functional images were normalized, spatially smoothed (Gaussian kernel with a 3-mm full-width half-maximum) and temporally high-pass filtered (3-dB cut-off = 100 s), and artifacts arising from head motion were removed using MCFLIRT^70^. Registration of task-related functional images to Montreal Neurological Institute space was performed in three stages: (1) the task-related limited-FOV echo-planar imaging (EPI) was registered to the presaturation whole-brain EPI using FMRIB’s Linear Image Registration Tool with six degrees of freedom transformation; (2) the whole-brain EPI was registered to the subject-specific structural images using boundary-based registration incorporating fieldmap correction^71^; (3) subject-specific structural images were registered to a 1-mm-resolution standard Montreal Neurological Institute template with FMRIB’s Non-linear Registration Tool^67^.

### fMRI data analysis

Statistical analysis of whole-brain functional data was performed at three levels using FMRIB’s Expert Analysis Tool^67,68^. In the first level, a univariate GLM was used to compute parameter estimates for each regressor in each session^72^. Contrast and variance estimate for each parameter in each participant were subsequently combined in a fixed-effects analysis conducted at the second level. Finally, a random-effects analysis was conducted at the third level, where subject identity was a random effect^73^. Significance testing was performed with cluster correction, a cluster significance threshold of *P* = . 001 and a voxel inclusion threshold of *z* = 3.1. Data were prewhitened before analysis to account for temporal autocorrelations in BOLD signal. We performed one whole-brain analysis for each of the four different approaches to computing the VoI (linear, UCB, symbolic and ANN). The GLM identified voxels where BOLD signal represented the value of staying, switching or selecting the attended or unattended patch:

18 $$\begin{array}{l}{\rm{BOLD}}=\beta 1\times {\rm{value}}\,{\rm{\_}}{\rm{of}}{\rm{\_}}{\rm{stay}}+\beta 2\times {\rm{value}}{\rm{\_}}{\rm{of}}{\rm{\_}}\,{\rm{switch}}\\ \,\,\,\,\,\,\,\,\,\,\,\,\,\,\,\,\,\,\,\,\,\,+\beta 3\times {\mu }_{{\rm{attended}}}+\beta 4\times {\mu }_{{\rm{unattended}}}+\beta 5\times {\rm{outcome}}+\epsilon \end{array}$$

All regressors were convolved with a double-gamma hemodynamic response function. Further nontask confound regressors were added to reduce noise in BOLD signal, including (1) head motion parameters estimated using MCFLIRT during preprocessing^70^; (2) regressors for voxel-wise estimates of physiological noise arising from cardiac and respiratory activity, estimated using FSL’s Physiological Noise Monitoring tool^74^; and (3) regressors for motion outliers, indicating volumes with head motion that could not be corrected with linear methods.

To compare the four different approaches to computing the VoI (linear, UCB, symbolic and ANN), we ran identical GLM analyses for each approach, varying only how the value of staying and switching were computed. For each of the 80 sessions (four sessions × 20 subjects), we fitted four separate GLMs using the value computations from each model. We then computed the MSE between the GLM predictions and the actual BOLD signal for each session, resulting in 80 MSE values per model. To assess whether the interpretable symbolic function performed comparably to the black-box ANN in predicting neural activity, we quantified effect sizes using Cohen’s *d* with 95% confidence intervals estimated via bootstrap resampling (10,000 iterations). This approach directly evaluates the magnitude of performance differences, which is critical for establishing whether the symbolic abstraction preserves the ANN’s predictive power while providing interpretability. To statistically compare the performance of the four approaches, we also conducted nonparametric Wilcoxon signed-rank tests on these paired MSE values, allowing us to assess whether one approach consistently provided better predictions of neural activity than the others. For ROI-based analyses, *P* values were Bonferroni-corrected for the number of ROIs tested (*n* = 5).

### ROI analysis

To analyze activity in specific ROIs (VTA, SN, DRN, LC, VSN), we extracted voxel-wise beta estimates (effect sizes) and their corresponding standard errors from the first-level GLM analysis for each relevant contrast (contrast of parameter estimates or ‘cope’ in FSL). For each ROI and contrast, we fitted a linear mixed-effects model using the MixedModels.jl package in Julia to estimate the group-level effect. The model formula was effsize ~ 1 + (1∣subject) + (1∣voxel), predicting the voxel-wise beta estimate (effsize) with a fixed intercept (representing the group effect) and random intercepts for subject and voxel to account for intersubject and intervoxel variability. Crucially, these models were weighted by the inverse variance of the beta estimates (1/standard_error^2^) to give more influence on more precise measurements at the voxel level. This approach yields a robust estimate of the average activation (the fixed intercept) for each contrast within each ROI across the group while appropriately accounting for different sources of variance. *P* values for the fixed intercept were extracted and corrected for multiple comparisons across the tested ROIs using the Bonferroni method.

### Reporting summary

Further information on research design is available in the Nature Portfolio Reporting Summary linked to this article.

## Online content

Any methods, additional references, Nature Portfolio reporting summaries, source data, extended data, supplementary information, acknowledgements, peer review information; details of author contributions and competing interests; and statements of data and code availability are available at 10.1038/s41593-026-02342-9.

## Supplementary information

Supplementary Information Supplementary Notes 1–7 (RSA analysis, lesion analysis of the symbolic equation, Markov decision process–based reference policy, end-to-end ANN benchmark, parameter recovery, feature selection, cross-task generalization), Figs. 1 and 2 (fMRI field of view, lesion analysis) and Tables 1 and 2 (per-ROI MSE model comparisons, model parameter descriptions).

Reporting Summary

## Source data

Source Data Fig. 2 Statistical source data underlying Fig. 2a,b.

Source Data Fig. 3 Statistical source data underlying Fig. 3b.

Source Data Fig. 4 Statistical source data underlying Fig. 4.

Source Data Fig. 5 Statistical source data underlying Fig. 5.

Source Data Fig. 6 Statistical source data underlying Fig. 6.

Source Data Extended Data Fig. 1 Statistical source data underlying Extended Data Fig. 1.

Source Data Extended Data Fig. 2 Statistical source data underlying Extended Data Fig. 2.

Source Data Extended Data Fig. 3 Statistical source data underlying Extended Data Fig. 3.

Source Data Extended Data Fig. 5 Statistical source data underlying Extended Data Fig. 5.

Source Data Extended Data Fig. 6 Statistical source data underlying Extended Data Fig. 6.

Source Data Extended Data Fig. 7 Statistical source data underlying Extended Data Fig. 7.

Source Data Extended Data Fig. 8 Statistical source data underlying Extended Data Fig. 8.

## Data Availability

All behavioral and neuroimaging data supporting the findings of this study are available via GitHub at https://github.com/simonedambrogio/HybridModellingProject/tree/main/Data. Source data are provided with this paper.

## Code availability

All analysis and modeling code is available via GitHub at https://github.com/simonedambrogio/HybridModellingProject and via Zenodo at 10.5281/zenodo.19685085 (ref. ^75^).
