# Supplementary material for: Interpretable abstractions of artificial neural networks predict behavior and neural activity during human information gathering
Source: Nat Neurosci. 2026 Jun 26;29(8):2036–47. doi: 10.1038/s41593-026-02342-9 (PMC13433322; doi:10.1038/s41593-026-02342-9)
Supplement: Supplementary file 1 [file 41593_2026_2342_MOESM1_ESM.pdf]

nature neuroscience

Supplementary information

https://doi.org/10.1038/s41593-026-02342-9

Interpretable abstractions of artificial neural networks predict behavior and neural activity during human information gathering

In the format provided by the authors and unedited

Supplementary Information

Simone D'Ambrogio, Jan Grohn, Nima Khalighinejad, Marcelo Mattar, Laurence Hunt, Matthew F. S. Rushworth

Manuscript (NN-T91487A): *Interpretable abstractions of artificial neural networks predict behavior and neural activity during human information gathering*

Contents

Supplementary Note 1 | Representational Similarity Analysis links AI and ACC multivariate activity to sampling behavior **1**

Supplementary Note 2 | Lesion analysis of the symbolic value-of-switching equation **2**

Supplementary Note 3 | MDP-based reference policy **2**

Supplementary Note 4 | End-to-end ANN benchmark **4**

Supplementary Note 5 | Parameter recovery of the ANN value-of-information function **4**

Supplementary Note 6 | Feature selection for the ANN value-of-information function **5**

Supplementary Note 7 | Cross-task generalization on external two-armed bandit datasets **5**

Supplementary Fig. 1 | fMRI field of view **7**

Supplementary Fig. 2 | Lesion analysis of the symbolic equation **8**

Supplementary Table S1 | Per-ROI MSE model comparisons **8**

Supplementary Table S2 | Model parameters and descriptions **10**

Supplementary Note 1 | Representational Similarity Analysis links AI and ACC multivariate activity to sampling behavior

Rationale. Our main-text analyses established that the ANN-derived value of information (VoI) predicts both sampling behavior and BOLD activity. This implies a crucial, though as yet untested, link: trial-by-trial variations in neural activity within VoI-encoding regions should directly correspond to trial-by-trial variations in sampling behavior. To explicitly test this implication and to investigate whether the representational structure of activity in these regions, beyond average activation levels, relates to behavior, we employed Representational Similarity Analysis (RSA). We reasoned that if a brain region computes decision variables guiding information sampling, then the similarity of its neural activity patterns across pairs of trials should mirror the similarity in the extent of sampling behavior observed on those same trials (Extended Data Fig. 8a). Based on our univariate results (Fig. 6a,b), we hypothesized that the neural patterns in AI and ACC would exhibit this correspondence with sampling duration.

Methods. We constructed Representational Dissimilarity Matrices (RDMs) for each participant and session (Extended Data Fig. 8b). A behavioral RDM captured the dissimilarity in sampling duration (number of samples taken) between pairs of trials using Euclidean distance. Neural RDMs were built from trial-level BOLD activation patterns (z-statistics from the first-level GLM), with the dissimilarity between any pair of trials defined as the Euclidean distance between their multi-voxel activation vectors. We focused on the four regions implicated in our univariate analyses (VTA, SN, AI, ACC), and to control for potential confounds related to ROI size and shape, we used masks matched to the VTA's shape, positioned at the center of each respective target region. Both behavioral and neural RDMs were vectorized by taking their upper-triangular elements (excluding the diagonal), and session-level Kendall's τ correlations were averaged within each participant per ROI before group-level testing.

Results. We calculated the Kendall's τ correlation between the flattened neural and behavioral RDMs for each session. Statistical testing across participants revealed significant positive correlations between neural pattern similarity and behavioral similarity in both the AI (two-sided Wilcoxon signed-rank test, $\tau = 0.024$, $P = 0.0006$, Bonferroni corrected $P = 0.0024$) and in the ACC ($\tau = 0.019$, $P = 0.0004$, Bonferroni corrected $P = 0.0017$), but not in the VTA ($\tau = 0.008$, $P = 0.072$) or SN ($\tau = 0.004$, $P = 0.46$, Bonferroni corrected $P = 1.0$) (Extended Data Fig. 8c). This suggests that the patterns of activity within AI and ACC, beyond their average activation levels, contain information related to the amount of information participants choose to sample on a given trial. The lack of significant correlation in VTA and SN, despite their univariate associations with decision variables, indicates that their trial-by-trial activity patterns may relate less directly to the specific duration of sampling compared to AI and ACC.

Supplementary Note 2 | Lesion analysis of the symbolic value-of-switching equation

Rationale and Results. One question arising from the symbolic regression is whether all components of the discovered equations are necessary. Specifically, the value of switching includes a constant and a logarithmic transformation: $\beta_3 + \exp(\beta_4 \log(2N_u)/N_a)$. We conducted a lesion analysis comparing three model variants: the original equation with $\log(2N_u)$; a simplified version without the constant "2", $\log(N_u)$; and an identity version without the logarithm and the constant (Supplementary Fig. 2). Removing the constant "2" did not impair model fit. In contrast, removing the logarithmic transformation substantially degraded performance across all participants. The logarithm thus captures an essential nonlinearity: diminishing sensitivity to evidence about the unattended option. As more samples accumulate, each additional sample provides proportionally less information about switching value—a compressive transformation that the linear (identity) function cannot capture.

Methods. We compared the three model variants using a bootstrapped 8-fold cross-validation procedure. Data were resampled with replacement to create 100 bootstrap samples per participant, and each sample was split into 8 folds. For each fold and variant, we performed 100 random parameter initializations and selected the best-fitting parameters. The loss metric was logit cross-entropy on held-out folds; models were optimized by gradient descent (4,000 iterations, gradient tolerance 10^{-6}). All three variants were fitted on identical cross-validation folds within each bootstrap sample, ensuring paired comparisons.

Supplementary Note 3 | MDP-based reference policy

Rationale. We computed a reference policy by solving the task as a Markov Decision Process (MDP). The resulting policy is *optimal* in the sense that it maximizes expected reward given the model's specific cost assumptions; it is not intended to represent uniquely correct human behavior. We use it only as a benchmark against which human and ANN-derived behavior can be compared.

State and action space. Each state s contains: the current time step, the number of revealed red dots in each patch, the total number of revealed dots in each patch, the current cursor position (left/right), and a binary visit indicator for each patch. The action space $\mathcal{A} = \{\text{stay, switch, select attended, select unattended}\}$. Selection terminates the episode.

Transition function. For sampling actions (stay/switch), the transition follows a Beta–Binomial with a uniform prior ($\alpha = \beta = 1$): given n revealed dots (r red, $n - r$ black) in the attended patch, the probability that the next dot is red is $(r + 1)/(n + 2)$ and black $(n - r + 1)/(n + 2)$.

Reward function. The reward $R(s,a)$ is:

$$R(s,a) = \begin{cases} -c_{\text{stay}}\,\Delta_n, & a = \text{stay} \\ -c_{\text{switch}}\,\Delta_n, & a = \text{switch} \\ p_A - (1-p_A), & a = \text{select attended} \\ (1-p_A) - p_A, & a = \text{select unattended} \end{cases} \tag{1}$$

where Δ_n is the per-step dot increment. The first sample of a trial is treated as a switch (the cursor enters a patch from the central start position). p_A is the probability that the true red-proportion of the attended patch exceeds that of the unattended patch; given posteriors $\text{Beta}(\alpha_k + 1, \beta_k + 1)$ for patch $k \in \{1, 2\}$, it is computed via a normal approximation:

$$p_A = \Phi\left(\frac{\hat{\mu}_1 - \hat{\mu}_2}{\sqrt{\hat{\sigma}_1^2 + \hat{\sigma}_2^2}}\right) \tag{2}$$

with $\hat{\mu}_k$ and $\hat{\sigma}_k^2$ the mean and variance of the posterior and Φ the standard normal CDF. To reflect the 13.3× ratio of switch-to-stay delay in the real task (≈2000 ms vs 150 ms), we set $c_{\text{switch}} = 13\,c_{\text{stay}}$ (specifically $c_{\text{stay}} = 0.005$, $c_{\text{switch}} = 0.065$).

Backwards induction. Because trials can start with different proportions of green-covered dots in each patch (range 0.05–0.3), we solved the MDP separately for 36 combinations of initial green-dot proportions (6 per patch: 0.1, 0.14, 0.18, 0.22, 0.26, 0.3). Parameters: maximum horizon = 100 steps, maximum dots per patch = 100, $\Delta_n = 2$, uniform prior $\alpha = \beta = 1$. The optimal value function was computed recursively:

$$V^*(s) = \max_{a \in \mathcal{A}} \left[R(s,a) + \sum_{s' \in \mathcal{S}'} T(s'|s,a)\, V^*(s') \right] \tag{3}$$

and the optimal policy selects the maximizing action at each state:

$$\pi^*(s) = \arg\max_{a \in \mathcal{A}} \left[R(s,a) + \sum_{s' \in \mathcal{S}'} T(s'|s,a)\, V^*(s') \right] \tag{4}$$

yielding a lookup table mapping each state to its optimal action, which we used to simulate MDP-derived behavior.

Supplementary Note 4 | End-to-end ANN benchmark

Rationale. To provide an upper bound on achievable predictive performance and to assess whether the interpretable cognitive structure sacrifices accuracy, we trained an end-to-end ANN that directly maps task states to action probabilities without imposing any cognitive scaffold.

Methods. The network takes as input 10 task-state features: counts of revealed dots in each patch (left, right, blocked), observed red proportions (left, right), initial uncertainty indicators

(green-covered dots in left and right patches), first-visit and after-first-visit indicators, and elapsed time. Continuous features were z-score normalized. The architecture consisted of three hidden layers (32, 32, and 16 neurons) with ReLU activations, followed by a linear output layer with 4 units corresponding to the four possible actions. The network was trained using the Adam optimizer to minimize logit cross-entropy loss over 3,000 iterations. Performance was evaluated using 8-fold cross-validation. To prevent overfitting, we evaluated validation loss after every training epoch and selected the model checkpoint with the lowest validation loss for each fold.

Supplementary Note 5 | Parameter recovery of the ANN value-of-information function

Rationale. To validate our hybrid modeling approach's ability to recover underlying value-of-information computations, we conducted a series of simulation studies.

Methods. We generated synthetic data from four different artificial agents, each using a distinct non-linear function to compute their value of information:

$$voi_1(n_A, n_U) = \sigma(0.94^{n_A - n_U}) - 0.2 \tag{5}$$

$$voi_2(n_A, n_U) = \sigma(0.94^{n_U - n_A}) - 0.2 \tag{6}$$

$$voi_3(n_A, n_U) = \sigma(0.94^{n_A - 37}) - 0.2 \tag{7}$$

$$voi_4(n_A, n_U) = \sigma(0.94^{37 - n_U}) - 0.2 \tag{8}$$

where σ represents the logistic function, and n_A and n_U represent the number of revealed dots in the attended and unattended patch respectively. These functions were chosen to represent different patterns of information valuation: the first two functions compute relative differences between patches, while the last two compare each patch against a fixed reference point. For each function, we simulated behavioral data using different sample sizes (4,000, 8,000, and 12,000 observations), chosen to span the range of samples observed in participant data (3,658–13,845; median = 8,339), to assess the robustness of our recovery procedure. We then applied our hybrid modeling approach to these simulated datasets, training the model to recover the underlying value-of-information function from the behavioral data alone. The recovered functions were compared to the true generating functions using Pearson correlation coefficients.

Supplementary Note 6 | Feature selection for the ANN value-of-information function

Rationale. To identify which task variables were essential for computing the value of information, we performed a systematic feature selection analysis.

Methods. We first defined a full feature vector containing eight input variables: cursor position (left/right), number of dots in the blocked option, number of dots in the left patch, number of dots in the right patch, whether it was the first visit to a patch, current cursor location, value of selecting the left patch (proportion of red dots), and value of selecting the right patch. We then conducted a leave-one-out analysis where we trained separate models for each participant, comparing the full model against versions with each feature individually removed. This resulted in eight different model variants per participant. All models were trained using 8-fold cross-validation. We evaluated the impact of each feature by comparing the validation performance of the reduced models against the full input-feature model. Features were considered critical if their removal led to a significant decrease in model performance across participants.

Supplementary Note 7 | Cross-task generalization on external two-armed bandit datasets

Rationale. To assess the generalizability of the insights derived from our modeling approaches, and to specifically compare the UCB model with the Symbolic model (derived from the symbolic regression analysis), we evaluated their performance on two additional, independent datasets from a previously published study by Gershman et al. (2018; referred to as "data1" and "data2" in our analyses, $N = 44$ and $N = 44$ respectively after excluding one subject from data1 that led to an extremely large value of loss when trying to fit the UCB model to their choices). These datasets involved similar information sampling tasks, providing a strong test for cross-task generalization. In this task, participants made sequential choices between two options across 20 blocks of 10 trials each, receiving stochastic reward feedback (drawn from Gaussian distributions) after each selection. Like our information sampling task, decisions depended on balancing exploration with exploitation based on accumulated evidence.

Models. For each participant in these external datasets, we fitted three models: the standard UCB model, an augmented UCB (AUCB) model, and the Symbolic model. All three models share a common structure: (1) a Q-learning component that updates expected value estimates based on observed rewards, (2) an exploration bonus that encourages sampling of uncertain options, and (3) a softmax function that converts values to choice probabilities. The Q-learning update for all models followed:

$$Q_{a,t+1} = Q_{a,t} + \alpha(r_t - Q_{a,t}) \tag{9}$$

where α is the learning rate, a is the chosen arm, and r_t is the observed reward. The total value

for each option was computed as $V = Q + U$, where U is the exploration bonus, which differed across models.

For the UCB model:

$$U_a = c\sqrt{\frac{\log(N)}{N_a}} \tag{10}$$

where c is a scaling parameter, N is the total number of samples from any option (equivalent to the trial number within a block), and N_a is the number of times arm a was chosen.

For the AUCB model:

$$U_a = c_0 + c_1\sqrt{\frac{\log(N)}{N_a}} \tag{11}$$

where c_0 provides a baseline offset. This AUCB model was included to control for the possibility that the Symbolic model's superior performance was due to its additional offset parameter rather than its functional form.

For the Symbolic model, the exploration bonus was computed separately for the chosen arm (a) and unchosen arm (u):

$$U_a = \beta_1 + \exp\left(-|\beta_2|\frac{N_a}{N_u}\right) \tag{12}$$

$$U_u = \beta_3 + \exp\left(-|\beta_4|\frac{\log(2N_u)}{N_a}\right) \tag{13}$$

Choice probabilities were computed via softmax:

$$P(a) = \frac{\exp(V_a/\tau)}{\sum_k \exp(V_k/\tau)} \tag{14}$$

where τ is the temperature parameter. This AUCB model has the same parametric flexibility as the core components of our Symbolic model, allowing for a more controlled comparison of functional forms.

Evaluation. To evaluate how well each model could predict each participant's choices, we employed a Leave-One-Out (LOO) cross-validation procedure on a per-subject basis. This involved iteratively training each model on all but one trial for a given subject and then testing its predictive accuracy on the held-out trial. This process was repeated such that every trial served as part of a test set. The primary metric for evaluating model performance was the average prediction loss (cross-entropy loss) on these held-out test trials, calculated for each subject for each model. The per-subject average LOO losses for the Symbolic and UCB models were then statistically compared to determine if one model offered consistently better predictions of choice behavior on these novel datasets. This comparison involved paired t-tests on the differences in mean losses per subject.

Supplementary Fig. 1 | fMRI field of view

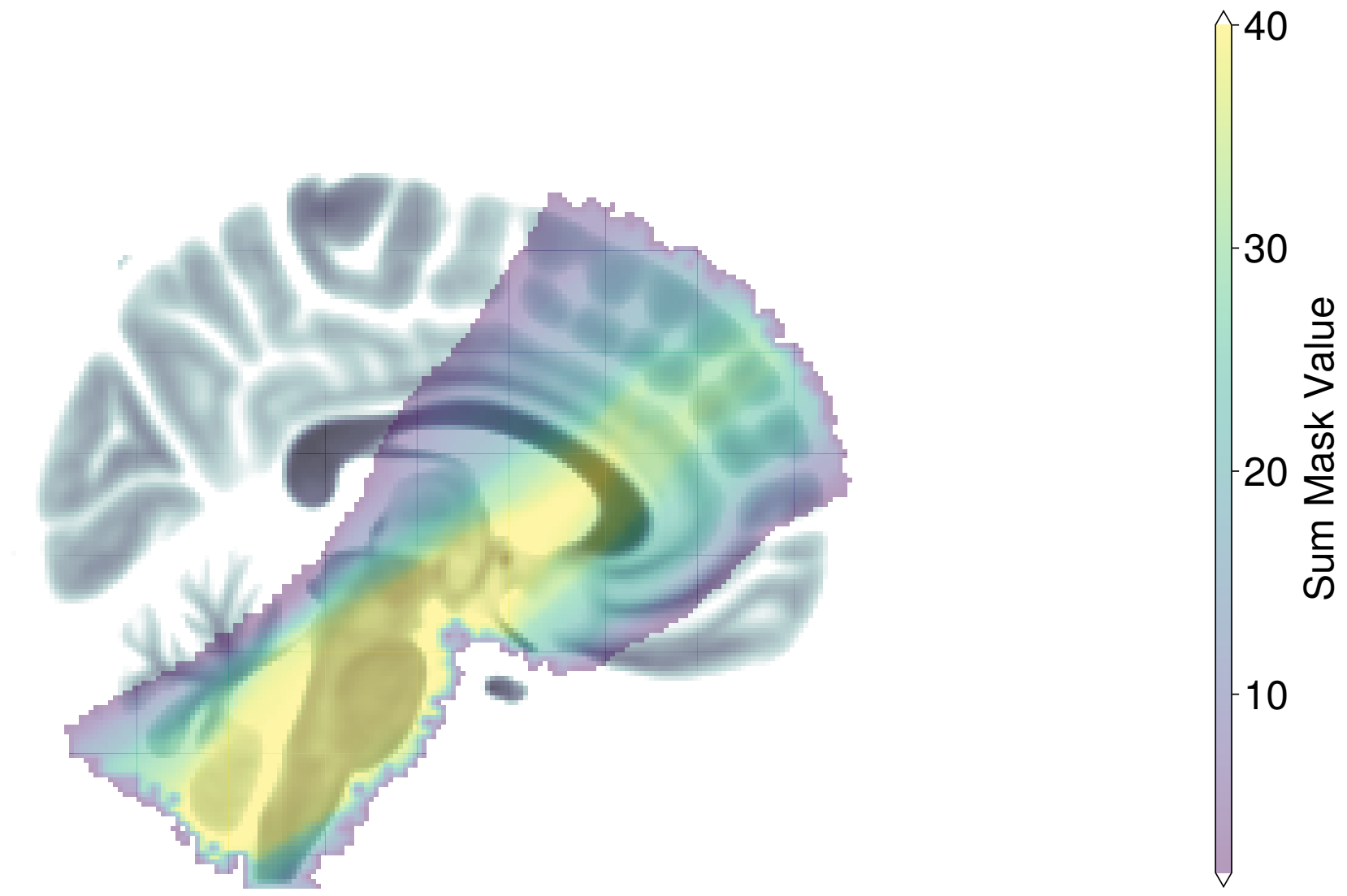

Supplementary Fig. 1 | Field of view coverage across sessions. Sagittal view ($x = 87$) showing the spatial coverage of the limited field of view functional imaging across all 40 sessions (2 sessions × 20 participants). The grayscale background shows the MNI152 T1 1 mm brain template. The colored overlay represents the sum of individual session masks, with the color scale indicating the number of sessions with coverage at each voxel location. Warmer colors indicate regions consistently captured across more sessions, while cooler colors represent areas with coverage in fewer sessions.

Supplementary Fig. 2 | Lesion analysis of the symbolic equation

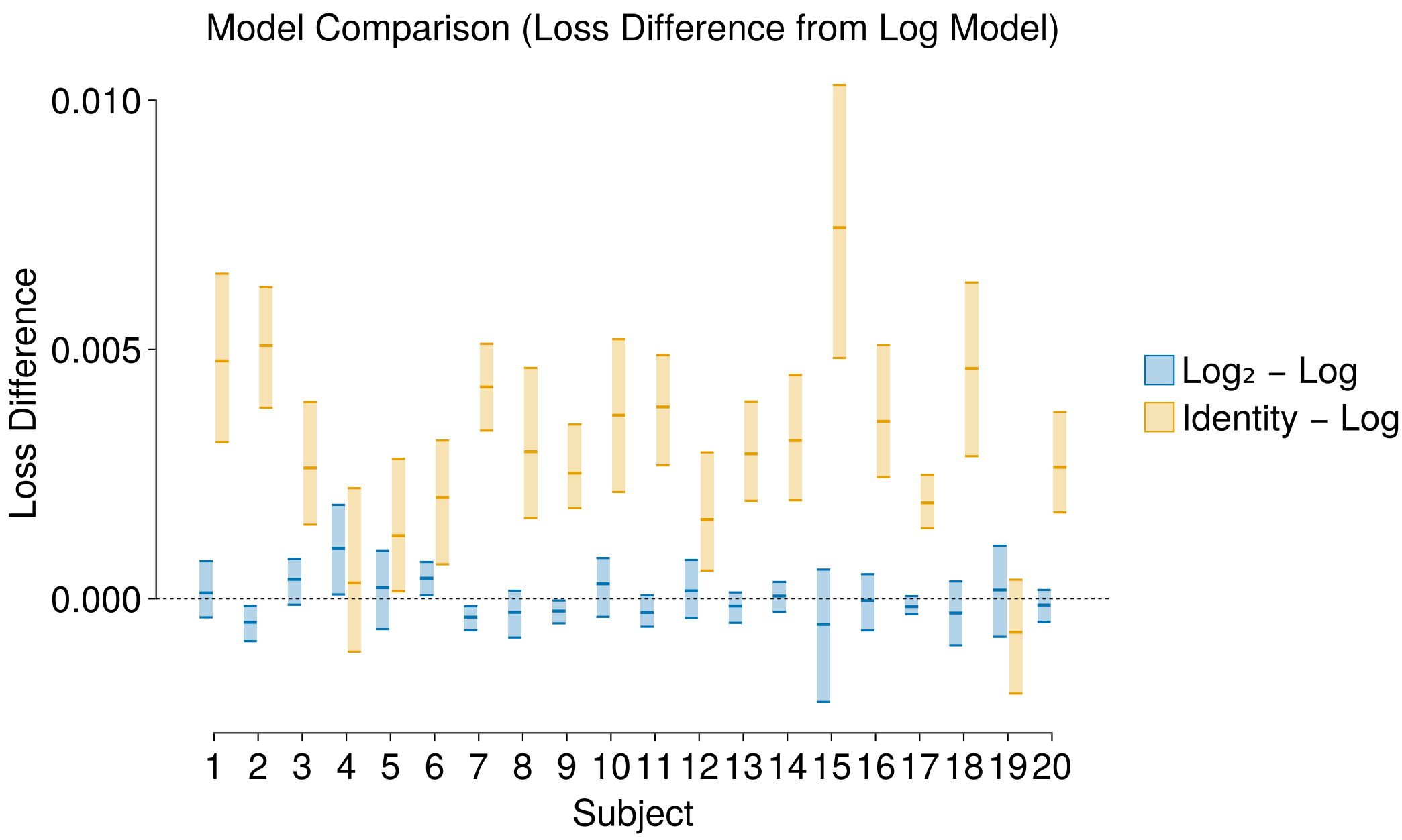

Supplementary Fig. 2 | Analysis of symbolic function components. Model comparison showing loss differences relative to the full model with $\log(2N_u)$ for each participant. Blue bars show the difference between the simplified model with $\log(N_u)$ and the full model (Log − Log2), centered near zero across all participants, indicating that removing the constant "2" does not impair model fit. Yellow bars show the difference between the identity model with N_u directly and the full model (Identity − Log), consistently positive across participants, indicating that removing the logarithmic transformation substantially degrades performance. Error bars represent 95% confidence intervals from 100 bootstrap iterations with 8-fold cross-validation.

Supplementary Table S1 | Per-ROI MSE model comparisons

Supplementary Table S1 | Model comparison for predicting neural activity across regions of interest. Cohen's d effect sizes and Wilcoxon signed-rank test P-values for pairwise model comparisons of per-voxel mean squared error in predicting BOLD activity. Negative d indicates the first model had lower MSE (better fit). Small effect sizes ($|d| < 0.2$) for ANN vs. Symbolic indicate comparable performance. $n = 80$ sessions (20 participants $\times$ 4 sessions). Two-sided Wilcoxon signed-rank tests; P-values for subcortical ROIs (VTA, SN, LC, DRN, VSN) are Bonferroni-corrected across the 5 subcortical ROIs; P-values for cortical ROIs (AI, ACC) are uncorrected.

	ANN vs. Sym		ANN vs. Lin		ANN vs. UCB		Sym vs. Lin		Sym vs. UCB	
**ROI**	*d*	*P*	*d*	*P*	*d*	*P*	*d*	*P*	*d*	*P*
Cortical										
AI	−0.06	0.814	−0.99	1.9×10^{-12}	−1.07	9.9×10^{-13}	−0.91	1.1×10^{-11}	−0.98	4.3×10^{-12}
ACC	−0.01	0.561	−0.22	6.9×10^{-5}	−0.14	1.4×10^{-5}	−0.20	6.1×10^{-5}	−0.11	1.5×10^{-4}
Subcortical (Bonferroni-corrected P, 5 ROIs)										
VTA	−0.11	1	−0.28	0.009	−0.34	0.003	−0.26	0.039	−0.33	0.007
SN	0.12	1	−0.33	<0.001	−0.33	<0.001	−0.38	<0.001	−0.36	<0.001
LC	−0.19	0.013	−0.28	0.041	−0.22	0.082	−0.19	0.485	−0.13	0.731
DRN	−0.23	0.008	−0.28	<0.001	−0.24	0.002	−0.21	0.108	−0.16	0.520
VSN	−0.14	0.021	−0.37	<0.001	−0.36	<0.001	−0.33	0.002	−0.32	0.001

Abbreviations: AI, anterior insula; ACC, anterior cingulate cortex; VTA, ventral tegmental area; SN, substantia nigra; LC, locus coeruleus; DRN, dorsal raphe nucleus; VSN, ventral septal nucleus. Lin, linear model; UCB, Upper Confidence Bound; ANN, artificial neural network model; Sym, symbolic model.

Supplementary Table S2 | Model parameters and descriptions

Supplementary Table S2 | Model parameters and descriptions. Free parameters of the four candidate models. Common cognitive-module parameters (λ_0, ω, κ_1, λ_2, τ) are shared across all value-of-information (VoI) variants. Model-specific parameters govern the VoI computation itself. Superscripts $^{(1)}$ and $^{(>1)}$ on Symbolic parameters denote separate fits for first visits and subsequent visits to a patch.

Model	Parameter	Count	Description
**All Models**	λ_0	1	**First-visit proportion estimate**: how the proportion of red dots observed in the attended patch influences the estimate of red dots in the unattended patch during first visit. $\lambda_0 = 0$ assumes 50%; $\lambda_0 = 1$ fully uses attended patch proportion.
	ω	1	**Interference resistance**: resistance to interference from blocked/ irrelevant options. Values closer to 1 indicate better ability to ignore task-irrelevant information.
	κ_1	1	**Switching cost**: cost of switching attention between patches. Higher values make switches more costly, promoting sustained attention.
	λ_2	1	**Memory decay rate**: rate at which information about unattended patches decays in memory over time. Higher values $\Rightarrow$ faster forgetting.
	τ	1	**Decision temperature**: stochasticity of action selection. Lower values make choices more deterministic, higher values more random.
**Linear**	β_1	1	**VoI offset**: baseline value of information, independent of evidence.
	β_2	1	**VoI slope**: linear scaling factor for how VoI changes with evidence.
	**Total**	**7**	**Linear form**: VoI $= \beta_1 + \beta_2 \times \mathit{evidence}$.
**UCB**	β	1	**Exploration bonus**: scaling factor for the Upper Confidence Bound exploration bonus (strength of uncertainty-driven exploration).
	**Total**	**6**	**UCB form**: VoI $= \beta\sqrt{\log(N)/n}$, where N is total evidence and n is evidence from the current patch.
**Symbolic**	$\beta_1^{(1)}$	1	**Attentional inertia (first visit)**: baseline value for continuing to sample from the current patch on first visit.
	$\beta_2^{(1)}$	1	**Information satiation (first visit)**: rate at which accumulated evidence reduces the desire to continue sampling during first visit.
	$\beta_3^{(1)}$	1	**Undirected exploration (first visit)**: baseline tendency to explore alternatives on first visit.
	$\beta_4^{(1)}$	1	**Directed exploration (first visit)**: rate at which learning about the current option increases interest in alternatives during first visit.
	$\beta_1^{(>1)}$	1	**Attentional inertia (subsequent visits)**: baseline value for continuing to sample from the current patch after first visit.
	$\beta_2^{(>1)}$	1	**Information satiation (subsequent visits)**: rate at which accumulated evidence reduces the desire to continue sampling after first visit.
	$\beta_3^{(>1)}$	1	**Undirected exploration (subsequent visits)**: baseline tendency to explore alternatives after first visit.
	$\beta_4^{(>1)}$	1	**Directed exploration (subsequent visits)**: rate at which learning about the current option increases interest in alternatives after first visit.
	**Total**	**13**	**Symbolic form**: Stay $= \beta_1 + \exp(-\lvert\beta_2\rvert\, N_a/N_u)$, Switch $= \beta_3 + \exp(-\lvert\beta_4\rvert \log(2N_u)/N_a)$.
**ANN**	θ	7,592	**Neural network weights**: parameters of the Lipschitz-Bounded Deep Network (4 hidden layers $\times$ 32 neurons each).
	**Total**	**7,597**	**Data-driven form**: VoI = LBDN(N_a/100, N_u/100, cursor, first-visit).
