# Supplementary material for: Interpretable abstractions of artificial neural networks predict behavior and neural activity during human information gathering
Source: Nat Neurosci. 2026 Jun 26;29(8):2036–47. doi: 10.1038/s41593-026-02342-9 (PMC13433322; doi:10.1038/s41593-026-02342-9)
Supplement: Supplementary file 2 [file 41593_2026_2342_MOESM2_ESM.pdf]

Corresponding author(s): Simone D'Ambrogio

Last updated by author(s): Jun 26, 2025

Reporting Summary

Nature Portfolio wishes to improve the reproducibility of the work that we publish. This form provides structure for consistency and transparency in reporting. For further information on Nature Portfolio policies, see our Editorial Policies and the Editorial Policy Checklist.

Statistics

For all statistical analyses, confirm that the following items are present in the figure legend, table legend, main text, or Methods section.

n/a	Confirmed	
☐	☒	The exact sample size (*n*) for each experimental group/condition, given as a discrete number and unit of measurement
☐	☒	A statement on whether measurements were taken from distinct samples or whether the same sample was measured repeatedly
☐	☒	The statistical test(s) used AND whether they are one- or two-sided<br>*Only common tests should be described solely by name; describe more complex techniques in the Methods section.*
☐	☒	A description of all covariates tested
☐	☒	A description of any assumptions or corrections, such as tests of normality and adjustment for multiple comparisons
☐	☒	A full description of the statistical parameters including central tendency (e.g. means) or other basic estimates (e.g. regression coefficient) AND variation (e.g. standard deviation) or associated estimates of uncertainty (e.g. confidence intervals)
☐	☒	For null hypothesis testing, the test statistic (e.g. *F*, *t*, *r*) with confidence intervals, effect sizes, degrees of freedom and *P* value noted<br>*Give P values as exact values whenever suitable.*
☒	☐	For Bayesian analysis, information on the choice of priors and Markov chain Monte Carlo settings
☒	☐	For hierarchical and complex designs, identification of the appropriate level for tests and full reporting of outcomes
☐	☒	Estimates of effect sizes (e.g. Cohen's *d*, Pearson's *r*), indicating how they were calculated

Our web collection on statistics for biologists contains articles on many of the points above.

Software and code

Policy information about availability of computer code

Data collection	jsPsych v6 was used to control the behavioural task, and acquire behavioural data.
Data analysis	Data analysis was performed using custom written code in Julia (v1.11) and python (v3.12.1)<br>Julia packages: ArgParse; BenchmarkTools; CairoMakie; CategoricalArrays; ColorSchemes; Colors; ComponentArrays; CSV; DataFrames; DataStructures; DelimitedFiles; Distributions; Flux; ForwardDiff; GLM; GLMakie; HypothesisTests; JLD2; JSON; LinearAlgebra; MixedModels; MLUtils; NNlib; OneHotArrays; Optim; ProgressBars; Random; RData; RobustNeuralNetworks; Statistics; StatsBase; StatsFuns; SymbolicRegression; YAML<br>Python packages: argparse; ast; glob; matplotlib; nibabel; numpy; os; pandas; pathlib; re; scipy; seaborn; subprocess; sys; yaml; fsl; simple_slurm

For manuscripts utilizing custom algorithms or software that are central to the research but not yet described in published literature, software must be made available to editors and reviewers. We strongly encourage code deposition in a community repository (e.g. GitHub). See the Nature Portfolio guidelines for submitting code & software for further information.

Data

Policy information about availability of data

All manuscripts must include a data availability statement. This statement should provide the following information, where applicable:

- Accession codes, unique identifiers, or web links for publicly available datasets
- A description of any restrictions on data availability
- For clinical datasets or third party data, please ensure that the statement adheres to our policy

Data is available at: https://github.com/simonedambrogio/HybridModellingProject/tree/main/Data

Research involving human participants, their data, or biological material

Policy information about studies with human participants or human data. See also policy information about sex, gender (identity/presentation), and sexual orientation and race, ethnicity and racism.

Reporting on sex and gender	*Use the terms sex (biological attribute) and gender (shaped by social and cultural circumstances) carefully in order to avoid confusing both terms. Indicate if findings apply to only one sex or gender; describe whether sex and gender were considered in study design; whether sex and/or gender was determined based on self-reporting or assigned and methods used.*<br>*Provide in the source data disaggregated sex and gender data, where this information has been collected, and if consent has been obtained for sharing of individual-level data; provide overall numbers in this Reporting Summary. Please state if this information has not been collected.*<br>*Report sex- and gender-based analyses where performed, justify reasons for lack of sex- and gender-based analysis.*
Reporting on race, ethnicity, or other socially relevant groupings	*Please specify the socially constructed or socially relevant categorization variable(s) used in your manuscript and explain why they were used. Please note that such variables should not be used as proxies for other socially constructed/relevant variables (for example, race or ethnicity should not be used as a proxy for socioeconomic status).*<br>*Provide clear definitions of the relevant terms used, how they were provided (by the participants/respondents, the researchers, or third parties), and the method(s) used to classify people into the different categories (e.g. self-report, census or administrative data, social media data, etc.)*<br>*Please provide details about how you controlled for confounding variables in your analyses.*
Population characteristics	*Describe the covariate-relevant population characteristics of the human research participants (e.g. age, genotypic information, past and current diagnosis and treatment categories). If you filled out the behavioural & social sciences study design questions and have nothing to add here, write "See above."*
Recruitment	*Describe how participants were recruited. Outline any potential self-selection bias or other biases that may be present and how these are likely to impact results.*
Ethics oversight	*Identify the organization(s) that approved the study protocol.*

Note that full information on the approval of the study protocol must also be provided in the manuscript.

Field-specific reporting

Please select the one below that is the best fit for your research. If you are not sure, read the appropriate sections before making your selection.

☐ Life sciences ☒ Behavioural & social sciences ☐ Ecological, evolutionary & environmental sciences

For a reference copy of the document with all sections, see nature.com/documents/nr-reporting-summary-flat.pdf

Behavioural & social sciences study design

All studies must disclose on these points even when the disclosure is negative.

Study description	Data are quantitative experimental data.
Research sample	A sample of 20 students, staff and public members in and around Oxford took part in the study. Participants took part in four sessions of the same task that took place in two different days to increase the sample size while keeping high data quality.The final sample covered 80 sessions but are presented as 20 data points (averaged within participants).
Sampling strategy	We used random sampling. No statistical methods were used to pre-determine sample sizes but our sample sizes are larger to those reported in previous publications (for example, see Wittman et al., 2016 Nature Communications; Meder et al., 2017 Nature Communications).
Data collection	For all four experimental sessions, participants performed the task inside an MRI scanner in a separate room to the researcher and radiographers. We recorded the data with a computer using JsPsych and a MRI scanner.

Timing	Data collection took place between March 2023 and June 2023
Data exclusions	Exclusion criteria included previous or current neurological or psychiatric disorder and contraindications that prohibited MRI scanning. One participant was excluded because exhibited excessive motion during the scan (N=1). This left a final sample of N=20.
Non-participation	Premature termination of the experimental procedure are stated in 'data exclusions'.
Randomization	Participants were not allocated into experimental groups

Reporting for specific materials, systems and methods

We require information from authors about some types of materials, experimental systems and methods used in many studies. Here, indicate whether each material, system or method listed is relevant to your study. If you are not sure if a list item applies to your research, read the appropriate section before selecting a response.

Materials & experimental systems

n/a	Involved in the study
☒	☐ Antibodies
☒	☐ Eukaryotic cell lines
☒	☐ Palaeontology and archaeology
☒	☐ Animals and other organisms
☒	☐ Clinical data
☒	☐ Dual use research of concern
☒	☐ Plants

Methods

n/a	Involved in the study
☒	☐ ChIP-seq
☒	☐ Flow cytometry
☐	☒ MRI-based neuroimaging

Plants

Seed stocks	*Report on the source of all seed stocks or other plant material used. If applicable, state the seed stock centre and catalogue number. If plant specimens were collected from the field, describe the collection location, date and sampling procedures.*
Novel plant genotypes	*Describe the methods by which all novel plant genotypes were produced. This includes those generated by transgenic approaches, gene editing, chemical/radiation-based mutagenesis and hybridization. For transgenic lines, describe the transformation method, the number of independent lines analyzed and the generation upon which experiments were performed. For gene-edited lines, describe the editor used, the endogenous sequence targeted for editing, the targeting guide RNA sequence (if applicable) and how the editor was applied.*
Authentication	*Describe any authentication procedures for each seed stock used or novel genotype generated. Describe any experiments used to assess the effect of a mutation and, where applicable, how potential secondary effects (e.g. second site T-DNA insertions, mosiacism, off-target gene editing) were examined.*

Magnetic resonance imaging

Experimental design

Design type	Participants were doing a task while being scanned. The design was event-related.
Design specifications	Each participant completed four MRI sessions, with each session lasting 25 minutes (the scanner continuously acquired data throughout each session). The number of trials varied per session as participants were free to complete as many trials as possible within the fixed time constraint, with trial completion dependent on individual sampling strategies and decision speeds. Each trial consisted of multiple phases: (1) trial initiation (free response time), (2) patch preview by clicking on each patch to reveal green-covered dots (free response time), (3) sampling phase initiation by clicking a central button (free response time), (4) information sampling phase lasting up to 24 seconds when two alternatives were available or 33 seconds when all three patches were available, with a 2-second initial delay upon hovering over a patch before information became available, followed by sequential revelation of grey-covered dots every 150ms, and (5) feedback presentation through a "duel" animation (1300ms) followed by outcome display (win/lose image, 1500ms). In 20% of trials, one of the three patches was randomly blocked after the initial preview phase. No explicit inter-trial intervals were specified, as the task was self-paced with participants initiating each new trial manually, allowing for natural breaks between trials based on individual pacing preferences.
Behavioral performance measures	We recorded choice behaviour (predictor selections and confidence judgments), reaction times and all button presses on each trial.

Acquisition

Imaging type(s)
Functional and structural images were collected.

Field strength
7 Tesla

Sequence & imaging parameters
Imaging data were acquired with a Siemens 7 Tesla MRI scanner using a multiband gradient echo T2-weighted echo planar imaging sequence with multiband acceleration factor 2 and GRAPPA acceleration factor 2. Functional scans were acquired with 1mm isotropic voxels, TE = 27ms, TR = 1.378s, and 90° flip angle. The parameters were selected to maximize signal-to-noise ratio in subcortical areas. To accommodate the high temporal and spatial resolution requirements, functional scans used a limited field of view oriented at 45 degrees with respect to the AC-PC line (36 slices per volume), capturing regions of interest in the midbrain, brainstem, and interconnected cortical areas. Before acquiring task-related functional data, a presaturation single-measurement, whole-brain functional scan was obtained with the same orientation to facilitate registration. Structural data were acquired using a T1-weighted MP-RAGE sequence with 0.7mm isotropic voxels, GRAPPA acceleration factor 2, TR = 2200ms, TE = 3.02ms, and inversion time (TI) = 1050ms. To correct distortions arising from magnetic field inhomogeneities, a fieldmap sequence was acquired with 2mm isotropic voxels, TR = 620ms, TE1 = 4.08ms, and TE2 = 5.1ms. Physiological noise effects were monitored using cardiac and respiratory recordings acquired at 50Hz with a BioPac MP160 device.

Area of acquisition
A limited field of view (FOV) was used rather than whole-brain acquisition. The acquisition area was specifically positioned to record simultaneously from key regions of interest in the midbrain, brainstem, and interconnected cortical areas. The FOV was oriented at 45 degrees with respect to the AC-PC line and comprised 36 slices. This positioning was strategically designed to capture all five ascending neuromodulatory nuclei (ventral tegmental area [VTA], substantia nigra [SN], dorsal raphe nucleus [DRN], locus coeruleus [LC], and ventral septal nucleus [VSN]) and two interconnected cortical regions (anterior insula [AI] and anterior cingulate cortex [ACC]) that project to these nuclei. The specific area of acquisition was determined based on the anatomical locations of these pre-defined regions of interest, which have been previously implicated in uncertainty processing and information sampling. The limited FOV approach was chosen to maximize spatial and temporal resolution for these specific subcortical and cortical targets rather than providing whole-brain coverage.

Diffusion MRI ☐ Used ☒ Not used

Preprocessing

Preprocessing software
Imaging data was analysed using FMRIB's Software Library (FSL:https://fsl.fmrib.ox.ac.uk/fsl/fslwiki). Preprocessing stages included motion correction, correction for spatial distortion by applying the fieldmap, brain extraction, high-pass filtering and spatial smoothing using using full-width half maximum of 2mm.

Normalization
Images were co-registered to an individuals' high-resolution structural image and then nonlinearly registered to the MNI template using 12 degrees of freedom. (Jenkinson and Smith, 2001).

Normalization template
The MNI152 template was used as is standard in FSL.

Noise and artifact removal
Preprocessing stages included motion correction, correction for spatial distortion by applying the fieldmap, brain extraction, high-pass filtering and spatial smoothing using full-width half maximum of 2mm.

Volume censoring
No volume censoring was used.

Statistical modeling & inference

Model type and settings
We used univariate models. First, we estimated contrasts for each subject and condition (social and non-social) separately with a first-level analysis using FSL FEAT. We used a second-level analysis to average across conditions within subjects (FIXED effects). Finally, we used FLAME 1 (mixed model) as a third level analysis to combine data across subjects.

Effect(s) tested
We conducted two complementary fMRI analyses to test for neural correlates of ANN-derived value of information signals during the information sampling task. First, we performed whole-brain GLM analyses to test for effects of: (1) the difference between value of staying and value of switching (Figure 6A), and (2) the sum of value of staying and switching (Figure 6B). These contrasts were designed to identify brain regions encoding the relative preference for maintaining versus switching attention, and the overall potential for information gain, respectively. Second, we conducted targeted ROI analyses in pre-defined neuromodulatory nuclei (VTA, SN, DRN, LC, VSN) to test for effects of four specific regressors: the sum and difference of the value of information, and the sum and difference of the value of selection (Figure 6C). The full GLM model included five main regressors: value_of_stay, value_of_switch, mu_attended (proportion of red dots in attended patch), mu_unattended (proportion of red dots in unattended patch), and outcome, with all regressors convolved with a double-gamma hemodynamic response function. Additional confound regressors included head motion parameters, physiological noise estimates, and motion outliers. We compared three different approaches to computing the value of information (linear, UCB, and ANN-derived) using identical GLM frameworks, varying only in the computational method for deriving the value signals.

Specify type of analysis: ☐ Whole brain ☐ ROI-based ☒ Both

Anatomical location(s)
VTA, SN, DRN, VSN, LC

Statistic type for inference

(See Eklund et al. 2016)

Cluster-based family-wise error correction was used. We performed analyses with a cluster-forming threshold of $p<0.001$ ($z>3.1$).

Correction

FWE was used ($p<0.05$)

Models & analysis

n/a	Involved in the study
☒	☐ Functional and/or effective connectivity
☒	☐ Graph analysis
☐	☒ Multivariate modeling or predictive analysis

Multivariate modeling and predictive analysis

We employed Representational Similarity Analysis (RSA) to test whether the similarity structure of neural activity patterns corresponded to the similarity structure of behavioral sampling duration on a trial-by-trial basis. Independent variables consisted of trial-level BOLD activation patterns (z-statistics from first-level GLM analyses) extracted from four regions of interest: ventral tegmental area (VTA), substantia nigra (SN), anterior insula (AI), and anterior cingulate cortex (ACC). Feature extraction involved creating multi-voxel activation vectors for each trial within anatomically-defined ROI masks. To control for potential confounds related to ROI size and shape differences, we used masks matched to the VTA's shape and positioned at the center of each target region. No explicit dimension reduction was applied to preserve the full representational structure of neural patterns.
The multivariate model constructed Representational Dissimilarity Matrices (RDMs) for both neural and behavioral data within each participant session. Neural RDMs captured the dissimilarity between BOLD activity patterns across pairs of trials using Euclidean distance, while behavioral RDMs captured the dissimilarity in sampling duration (number of samples taken) between the same trial pairs, also using Euclidean distance. Both RDMs were vectorized by extracting their upper triangular elements (excluding the diagonal) to create feature vectors for correlation analysis.
Training and evaluation involved computing Kendall's τ correlation coefficients between the vectorized neural and behavioral RDMs for each session. These session-level correlations were averaged within each participant for each ROI. Model performance was evaluated using one-sample Wilcoxon Signed Rank Tests across participants, testing for significant positive correlations between neural pattern similarity and behavioral similarity. P-values were Bonferroni corrected for multiple comparisons across the four tested ROIs, with significance threshold set at $P < 0.01$ after correction.
